# Assessing respiratory pathogen communities in bighorn sheep populations: Sampling realities, challenges, and improvements

**DOI:** 10.1371/journal.pone.0180689

**Published:** 2017-07-14

**Authors:** Carson J. Butler, William H. Edwards, Jessica E. Jennings-Gaines, Halcyon J. Killion, Mary E. Wood, Douglas E. McWhirter, J. Terrill Paterson, Kelly M. Proffitt, Emily S. Almberg, P. J. White, Jay J. Rotella, Robert A. Garrott

**Affiliations:** 1 Fish and Wildlife Ecology and Management Program, Department of Ecology, Montana State University, Bozeman, Montana, United States of America; 2 Wildlife Health Laboratory, Wyoming Game and Fish Department, Laramie, Wyoming, United States of America; 3 Wyoming Game and Fish Department, Laramie, Wyoming, United States of America; 4 Wyoming Game and Fish Department, Cody, Wyoming, United States of America; 5 Montana Department of Fish, Wildlife and Parks, Bozeman, Montana, United States of America; 6 Wildlife Health Laboratory, Montana Department of Fish, Wildlife and Parks, Bozeman, Montana, United States of America; 7 Yellowstone Center for Resources, Yellowstone National Park, National Park Service, Mammoth, Wyoming, United States of America; University of Illinois at Urbana-Champaign, UNITED STATES

## Abstract

Respiratory disease has been a persistent problem for the recovery of bighorn sheep (*Ovis canadensis*), but has uncertain etiology. The disease has been attributed to several bacterial pathogens including *Mycoplasma ovipneumoniae* and *Pasteurellaceae* pathogens belonging to the *Mannheimia*, *Bibersteinia*, and *Pasteurella* genera. We estimated detection probability for these pathogens using protocols with diagnostic tests offered by a fee-for-service laboratory and not offered by a fee-for-service laboratory. We conducted 2861 diagnostic tests on swab samples collected from 476 bighorn sheep captured across Montana and Wyoming to gain inferences regarding detection probability, pathogen prevalence, and the power of different sampling methodologies to detect pathogens in bighorn sheep populations. Estimated detection probability using fee-for-service protocols was less than 0.50 for all *Pasteurellaceae* and 0.73 for *Mycoplasma ovipneumoniae*. Non-fee-for-service *Pasteurellaceae* protocols had higher detection probabilities, but no single protocol increased detection probability of all *Pasteurellaceae* pathogens to greater than 0.50. At least one protocol resulted in an estimated detection probability of 0.80 for each pathogen except *Mannheimia haemolytica*, for which the highest detection probability was 0.45. In general, the power to detect *Pasteurellaceae* pathogens at low prevalence in populations was low unless many animals were sampled or replicate samples were collected per animal. Imperfect detection also resulted in low precision when estimating prevalence for any pathogen. Low and variable detection probabilities for respiratory pathogens using live-sampling protocols may lead to inaccurate conclusions regarding pathogen community dynamics and causes of bighorn sheep respiratory disease epizootics. We recommend that agencies collect multiples samples per animal for *Pasteurellaceae* detection, and one sample for *Mycoplasma ovipneumoniae* detection from at least 30 individuals to reliably detect both *Pasteurellaceae* and *Mycoplasma ovipneumoniae* at the population-level. Availability of PCR diagnostic tests to wildlife management agencies would improve the ability to reliably detect *Pasteurellaceae* in bighorn sheep populations.

## Introduction

Respiratory disease has been a persistent problem for bighorn sheep restoration, with mortality during epizootics ranging from 10% to 90% of the affected population [1]. Epizootics affecting all age classes are often followed by multiple years of depressed lamb recruitment [2,3] as well as additional all-age epizootics of varying duration and severity [3,4]. The episodic nature of these disease outbreaks has led to hypotheses regarding the role of resident pathogens in a population versus the periodic introduction of novel pathogens [5,6]. Rigorous testing of these hypotheses has been limited by difficulties in accurately characterizing pathogen communities hosted by populations both before and after disease epizootics begin [6]. In addition, the poly-microbial nature of respiratory disease has made the identification of causative agents a challenging task. Recent research suggests *Mycoplasma ovipneumoniae* and *Pasteurellaceae* family pathogens (leukotoxigenic strains of *Mannheimia* or *Bibersteinia* genus organisms and potentially *Pasteurella multocida*) can play a role in the development of respiratory disease in bighorn sheep [7–11].

Wildlife managers regularly invest resources towards sampling bighorn sheep populations to assess which respiratory pathogens are present. Presence of respiratory pathogens in live animals is typically assessed by swabbing their nasal cavity and tonsillar crypts or oropharynx and testing those swabs for pathogens using culture or polymerase chain reaction (PCR) methods. The results of these tests are used by wildlife managers to determine herd-health, disease-risk, and translocation decisions[12,13]. However, the detection probabilities (analogous to test sensitivity) of diagnostic protocols (i.e., the complete process by which samples are collected, handled, stored, and subjected to diagnostic tests) for live-sampled bighorn sheep may be low, particularly for *Pasteurellaceae* bacteria [14–16]; detection probabilities for *M*. *ovipneumoniae* diagnostic protocols have not been reported. Low detection probability has strong potential to lead to inaccurate conclusions about which pathogens are responsible for respiratory disease epizootics, and whether the outbreak was caused by introduction of novel pathogens or increased expression of pathogens already resident in the population (e.g., increased virulence or abundance within the host-pathogen complex, increased transmission rates, or increased proportion of susceptible individuals in the population) [5,17]. Additionally, low detection probability can lead to inappropriate or ineffective management decisions (e.g., translocations) when sampling does not detect important respiratory pathogens present in a bighorn sheep population.

The primary objectives of this study were to: 1) estimate detection probabilities for five bighorn sheep respiratory pathogens using various diagnostic protocols; 2) assess the precision with which respiratory pathogen prevalence can be estimated; 3) compare differences in prevalence estimates when detection error was, or was not, considered; and 4) evaluate the power to detect each pathogen in a population using different protocols with varying levels of pathogen prevalence, sampling intensity, and population sizes. Our goal is to provide guidance for sampling bighorn sheep respiratory pathogens and insights regarding the potential for mischaracterization of pathogen communities in bighorn sheep populations.

## Materials and methods

### Ethics statement

Capture and handling of animals reported herein comply with scientific guidelines and permits acquired from the State of Montana and the State of Wyoming. All animal capture and handling protocols were approved by Institutional Care and Use Committees at Montana State University (Permit # 2011–17, 2014–32), Montana Department of Fish, Wildlife, and Parks (Permit # 2016–005) or Wyoming Game and Fish Department (Permit # 854).

### Sample collection and diagnostic protocols

One to four tonsil and nasal swabs were collected by trained personnel from bighorn sheep sampled in nine free-ranging populations in Montana, ten free-ranging populations in Wyoming, and one captive population in Wyoming between March 2013 and March 2016. Animals were captured on publically or privately owned land between October 1 and March 31^st^ each year using chemical immobilization, baited drop-nets, or helicopter net-gunning. Animals captured via chemical immobilization were anesthetized using BAM ^™^ (27.3 mg Butorphanol, 9.1mg Azaperone, 10.9 mg Medetomidine per animal) and anesthetization was reversed using Tolazoline and Atipamezole. Animals that were sampled rarely showed evidence of active respiratory infection upon sampling, although some animals with runny noses or mild coughs were occasionally sampled.

Swab samples were collected using sterile polyester tipped applicators (Puritan #25–806 1PD, Guilford, Maine, USA) and were collected following methods recommended by the Western Association of Fish and Wildlife Agencies[12]. Samples were typically collected within five minutes of capture; however, in rare instances samples were collected up to 30 minutes following initial capture. Extreme care was taken with both swab types to avoid contamination caused by contacting non-target areas. Collection of tonsil swabs was aided by the use of a lighted swab extender and tongue depressors to better target the tonsils and tonsillar crypts. Both tonsils were targeted for each “tonsil swab sample”; however when animal resistance hindered access to the tonsils, a single tonsil was typically swabbed. Collection of nasal swabs entailed inserting an applicator 8–12 cm into each nostril and rotating the shaft. Tonsil swab samples were assessed for the presence of *Pasteurellaceae* pathogens and nasal swabs were assessed for presence of *M*. *ovipneumoniae*, with the exception of one sampling occasion where nasal swabs were also assessed for presence of *Pasteurellaceae* pathogens. Replicate sampling of individual animals was generally conducted using different diagnostic protocols, but was conducted by repeating the same protocol twice for a subset of individuals. Replicate tonsil swab samples from the same animal were collected individually and sequentially. Replicate nasal swab samples from animals sampled in Montana were also collected individually and sequentially, while replicate nasal swabs samples from animals sampled in Wyoming were collected in tandem (i.e., two applicators inserted into the nasal cavity together). For a subset of 106 animals, the sequence that protocols were conducted was systematically assigned and recorded to assess whether detection probability declined as samples were sequentially collected (see S1 Appendix). Samples were labeled so that replicates from the same individual animal could not be identified by laboratory personnel. The same set of diagnostic protocols was not used for all sampling occasions due to limited availability of trained personnel, specialized equipment, or transport media.

All *Pasteurellaceae* pathogens were detected using one set of five diagnostic protocols and *M*. *ovipneumoniae* was detected using a different set of three diagnostic protocols (Table 1). Diagnostic tests offered by a fee-for-service (FFS) laboratory (Washington Animal Disease Diagnostic Laboratory-WADDL) were used to detect and identify respiratory pathogens for four protocols (FFS protocols). Diagnostic tests conducted at a non-FFS diagnostic laboratory (Wyoming Game and Department Wildlife Health Laboratory-WGFD) were used to detect and identify respiratory pathogens for three protocols (non-FFS protocols). A non-FFS diagnostic test also was conducted at WADDL as part of protocol development. All *Pasteurellaceae* FFS protocols detected pathogens by culture while the *M*. *ovipneumoniae* FFS protocol used PCR. Non-FFS protocols used PCR (sometimes in conjunction with culture) to detect each pathogen, with the exception of *P*. *multocida*, which was only detected by culture.

**Table 1:** Summary of diagnostic protocols used in this study to detect respiratory pathogens in bighorn sheep.

Pathogen Group	Protocol	Media^1^	Diagnostic Lab^2^	Diagnostic Test^1^
*Pasteurellaceae*				
TSB^3^		TSB	WADDL	Culture
Port-A-Cul		Port-A-Cul^™^	WADDL	Culture
Plated Culture		CBA^4^ + TSB	WADDL	Culture
Plated PCR^5^		CBA	WGFD	PCR
Wyoming		CBA & Port-A-Cul^™^	WGFD	Culture + PCR
*Mycoplasma ovipneumoniae*				
TSB		TSB	WADDL	PCR
qPCR		None	WADDL	PCR
Wyoming		Port-A-Cul^™^ + TSB-1	WGFD	PCR

^1.^ Where two media or test types are listed, a “&” symbol indicates both where employed simultaneously and independently and a “+” symbol indicates a sequence. TSB is an abbreviation for tryptic soy broth with 15% glycerol, CBA is an abbreviation for Columbia Blood Agar ^™^, and TSB-1 is an abbreviation for modified tryptone soy broth.

^2.^ All protocols where samples were tested at WADDL, except *Mycoplasma ovipneumoniae* qPCR, were considered “fee-for-service protocols”, and all protocols where samples were tested at WGFD were considered “non fee-for-service”.

^3.^ TSB is abbreviation for tryptic soy broth

^4.^ CBA is abbreviation for Columbia blood agar.

^5.^ The Plated PCR protocol did not assess presence of *Pasteurella multocida*.

#### *Pasteurellaceae* protocols

*Pasteurellaceae* pathogens assessed in this study included beta-hemolytic or leukotoxigenic strains of *Bibersteinia trehalosi (B*. *trehalosi)*, *Mannheimia haemolytica (M*. *haemolytica)*, and *Mannheimia* spp., as well as *Pasteurella multocida (P*. *multocida)*. Presence of *Pasteurellaceae* pathogens was assessed using five diagnostic protocols explained below and described in detail in S2 Appendix.

Wyoming protocol: Two tonsil swabs were collected from each animal, one was used to immediately inoculate a Columbia Blood Agar (CBA) culture plates with 5% sheep blood (Hardy Diagnostics, Santa Maria, California, USA), and one was stored at approximately 4°C in a 10 mL Port-A-Cul^™^ transport media tube or a 10 mL Amies media without charcoal tube for approximately 6 hours before inoculating a second CBA plate which was incubated at 37°C in 5% CO_2_. *Pasteurellaaceae* pathogens on CBA plates were identified at WGFD using a combination of culture and PCR tests. Presence of *P*. *multocida* was assessed solely by culture.

TSB protocol: Swabs were each placed into a vial of tryptic soy broth with 15% glycerol (TSB; Hardy Diagnostics, Santa Maria, California, USA) immediately after collection. The TSB vials were immediately frozen (approximately -20°C), and later shipped overnight on dry ice to WADDL where they were assessed for presence of any *Pasteurellaceae* pathogens using FFS culture tests.

Port-A-Cul protocol: Swabs were immediately placed into 10 ml Port-A-Cul ^™^ transport media tubes (BD, Sparks, Maryland, USA), and kept cool until being shipped on ice overnight to WADDL within 48 hrs. Samples were assessed immediately upon arrival at WADDL for presence of any *Pasteurellaceae* pathogens using FFS culture tests.

Plated Culture protocol: Tonsil swabs were used to immediately inoculate a CBA plate which was then incubated at 37°C in 5% CO_2_. After 24 hours, a swab of the primary streak zone and phenotypically distinct (though unidentified) colonies was collected from each CBA plate, placed into TSB, and immediately frozen at approximately -20°C. Vials were shipped overnight on dry ice to WADDL where they were assessed for presence of any *Pasteurellaceae* pathogens using FFS culture tests.

Plated PCR protocol: Following completion of the Plated Culture protocol, the inoculated CBA plates were incubated for approximately 24 additional hours at 37°C in 10% CO_2._ Then bacterial growth was removed from the surface of the CBA plate, suspended in 4ml of PBS and frozen at -20°C until PCR tests were conducted at WGFD. Presence of *P*. *multocida* was not assessed by this protocol.

#### *Mycoplasma ovipneumoniae* protocols

Presence of *M*. *ovipneumoniae* was assessed using the three diagnostic protocols explained below and described in detail in S2 Appendix:

Wyoming Protocol: Swabs were immediately placed into a 10 mL Port-A-Cul^™^ transport media tube or Amies media without charcoal in 10 mL culture tubes and kept at approximately 4°C until being placed in tryptone soy broth (TSB-1) [18] with several modifications [19] within 72 hours of collection. The modified TSB-1 broth was incubated at 37°C in 5% CO_2_ for 48 hours and tested for presence of *M*. *ovipneumoniae* at WGFD using PCR.

TSB protocol: Swabs were immediately placed into TSB vials after sample collection. The TSB vials were immediately frozen (approximately -20°C), and later shipped overnight on dry ice to WADDL where they were assessed for *M*. *ovipneumoniae* presence using an FFS PCR test.

qPCR protocol: Swabs were immediately sealed in a sterile 4mL or 2mL vial after sample collection and kept at approximately -20°C. Vials were later shipped overnight on dry ice to WADDL where they were tested for *M*. *ovipneumoniae* using quantitative PCR (qPCR) as part of new protocol development (i.e., this is not an FFS diagnostic test).

### Pathogen classification

*Pasteurellaceae* organisms were classified based on hemolysis on CBA or the presence of the leukotoxin gene (*lktA*) as indicated by PCR; non-hemolytic/non-leukotoxigenic strains in the *Mannheimia* or *Bibersteinia* genera were not considered in this analysis. To make results obtained from culture and PCR tests comparable, beta-hemolysis and presence of *lktA* were considered synonymous in our categorization of organisms, as beta-hemolysis in culture is well correlated with presence of the *lktA* gene in *Pasteurellaceae* organisms [20]. It should be noted, however, that presence of the *lktA* gene indicates potential virulence of an organism, but does not necessarily indicate that leukotoxin is being actively produced by the organism [21]. Diagnostic tests do not consistently distinguish among some species in the *Mannheimia* genus [22]. For this analysis *Mannheimia glucosida* was included within the *M*. *haemolytica* classification, as the available PCR primers amplify the target genes of both species [8]. Other pathogens in the *Mannheimia* genus, including *Mannheimia ruminalis* and unidentified *Mannheimia* spp., were combined into a single group for analysis (*Mannheimia* spp.), as isolates currently identified as *M*. *ruminalis* by WADDL were reported as unidentified *Mannheimia species* at the beginning of this study. Additionally, the WGFD diagnostic tests had insufficient specificity to distinguish *M*. *ruminalis* from other species in the *Mannheimia* genus. *B*. *trehalosi*, *P*. *multocida* and *M*. *ovipneumoniae* were classified as reported by the diagnostic laboratory.

### Estimating detection probability and prevalence

Single-species, single-season occupancy modeling [23] using package “unmarked” [24] in program R [25] was used to independently estimate detection probability (ρ) and prevalence (ψ) of each respiratory pathogen within each population, where individual bighorn sheep constituted sampling sites and the protocols that were conducted constituted the encounter history for each animal. For each pathogen, estimates of detection probability for each protocol were obtained by running a single complex model structure to incorporate as many sources of variability as our data would allow. This model allowed detection probability to vary by protocol and allowed prevalence to vary by population and year. Assumptions of this modeling approach include: (1) the infection status of an animal does not change during the sampling period; (2) all animals have the same probability of the pathogen being present or heterogeneity is accounted for; (3) the probability of a positive detection from a sample is the same for all animals that host the pathogen or heterogeneity is accounted for; (4) detections are independent; and (5) there are no false positive detections. These assumptions are discussed in S1 Appendix.

To reduce the number of model parameters and avoid model-convergence issues related to estimating logit-scaled parameters at a boundary, data from population-years where a respiratory pathogen was undetected and/or fewer than five animals were sampled were omitted from analysis as they provide no information related to detection probability. Adequate model convergence was confirmed by verifying that each model’s condition number was less than 10^4^ [26]. An attempt was made to estimate overdispersion (*ĉ*) by applying the MacKenzie and Bailey goodness-of-fit test [27] to the full model (ρ ~ Protocol, *Prevalence* ~ Population-Year) for each pathogen; however, the expected values that generate the χ^2^ statistics for this test were often less than one, indicating that the data were too sparse to accurately estimate *ĉ* [28].

Several of the diagnostic protocols were conducted in just one of the states (Montana or Wyoming) where we sampled bighorn sheep. As a result, the Wyoming Protocol was never conducted in the same population as the Plated Culture or Plate PCR protocols for *Pasteurellaceae*. This weakness in study design could have led to inaccurate estimates of detection probability for all protocols if detection probability of our ubiquitously-used protocol (TSB Protocol) differed between the states. Therefore, it was important to assess whether estimates of detection probability for our ubiquitously-used protocol (TSB Protocol) differed when conducted in different states. Evidence for differences in detection probability estimates for each pathogen was assessed by fitting the full model to two subsets of data collected by different personnel in different states (Montana or Wyoming) and comparing the resulting estimates of detection probability to those obtained from analysis of the complete dataset. A complete description of this assessment can be found in S3 Appendix.

### Assessing power to detect pathogens at the population-level

Estimates of pathogen detection probability for different protocols can be used to estimate the minimum sampling effort required to determine presence of respiratory pathogens in a population. In this context, the power of a protocol to detect a pathogen in an infected bighorn sheep population (i.e., the probability of detecting a pathogen in at least one animal) can be derived from the estimate of detection probability for that protocol. A hierarchical model was developed to derive the power to detect each pathogen in a population of bighorn sheep given the protocol used, number of animals sampled and number of times the protocol was conducted per animal, pathogen prevalence, and population size. A full description of the derivation is provided in S4 Appendix.

The power to detect each of the pathogens in at least one animal in a population during a sampling occasion was calculated (given the estimate of detection probability and associated standard error for a specified pathogen and protocol) while varying the number of animals sampled (from 1 to 100), the number of times the protocol was conducted per animal (1, 2 or 3), pathogen prevalence (0.10, 0.30, or 0.50), and population size (25, 50, 100, or 200). These findings were used to identify sampling methodologies (i.e., combinations of diagnostic protocols, animals sampled, and number of times the protocols are conducted per animal) predicted to result in adequate detection power for each pathogen (defined as ≥0.80 following Ellis *et al*. 2014 [29]). To illustrate the effects of specific variables on detection power variables were constrained to subjective “default” values. Unless otherwise specified, the “default” number of animals sampled was set at 25, population size was set at 100, number times a diagnostic protocol was conducted per animal was one, and the default protocol was the TSB protocol. Default prevalence was set at 0.10, which was considered a realistic value to represent low prevalence based on estimates obtained in this study. Protocols whose detection probability was estimated to be zero or one (*Mannheimia* spp.-Port-A-Cul protocol, *B*. *trehalosi*- Port-A-Cul protocol, *Pasteurella multocida*-Plated Culture protocol) were not considered in this assessment.

## Results

### Sampling effort

A total of 2093 *Pasteurellaceae* diagnostic tests were conducted for 476 bighorn sheep and a total of 768 *M*. *ovipneumoniae* diagnostic tests were conducted for 469 bighorn sheep. The TSB and Plated Culture (*Pasteurellaceae)* protocols were conducted twice for 165 and 61 animals, respectively; all other *Pasteurellaceae* protocols were conducted once per animal. Various combinations of two *Pasteurellaceae* protocols were conducted on 178 animals; three *Pasteurellaceae* protocols were conducted on 108 animals; four *Pasteurellaceae* protocols were conducted on 23 animals; five *Pasteurellaceae* protocols were conducted on 26 animals; and six *Pasteurellaceae* protocols were conducted on 45 animals. Among *M*. *ovipneumoniae* protocols, only the TSB protocol was conducted more than once per animal, and was conducted twice for 117 animals. Various combinations of two *M*. *ovipneumoniae* protocols were conducted on 278 animals and three *M*. *ovipneumoniae* protocols were conducted on 11 animals.

### Pathogen detection summaries

The total number of detections for individual pathogen species varied from 44 *P*. *multocida* detections to 152 *M*. *ovipneumoniae* detections (Table 2). Considering animals where multiple diagnostic protocols were conducted and the respective pathogens were detected, *M*. *haemolytica* was detected by more than one diagnostic test 30% of the time (14 of 46 animals), *Mannheimia* spp. 7% (9 of 128), *B*. *trehalosi* 37% (22 of 60), *P*. *multocida* 2% (1 of 42), and *M*. *ovipneumoniae* 60% of the time(35 of 59). When the targeted pathogens were detected in a population-year, the minimum estimates of naïve prevalence (i.e., the proportion of animals from a population where the pathogen was detected in a given year) varied from 0.03 to 0.17 among the pathogens and the estimates of maximum naïve prevalence varied from 0.44 to 1.00 (Table 2).

**Table 2:** Sampling results summaries and naïve prevalence estimates for five bighorn sheep respiratory pathogens.

	*Mycoplasma ovipneumoniae*	*Mannheimia haemolytica*^1^^,^^2^	*Mannheimia spp*.^2^	*Bibersteinia trehalosi*^2^	*Pasteurella multocida*
Diagnostic tests^3^ (# Positive)	768(152)	1268(82)	1268(141)	1268(94)	1057(44)
Animals sampled (# Positive)	469(118)	476(66)	476(132)	476(67)	476(43)
Range of naïve prevalence^4^	0.051–0.828	0.062–0.440	0.167–0.615	0.029–1.000	0.059–0.875

^1.^This classification includes isolates identified as *Mannheimia glucosida* (n = 8).

^2.^Only beta-hemolytic or leukotoxigenic strains are summarized.

^3.^The *total* number of individual *Pasteurellaceae* diagnostic tests is 2093, not the apparent sum, because an individual culture test assesses presence of all four targeted *Pasteurellaceae* pathogens.

^4.^Naïve prevalence is estimated as the proportion of animals from a sampled population in a given capture season in which the pathogen was detected. Population-years where less than 5 animals were sampled and where a pathogen was not detected are not included.

### Detection probability estimates

**Fig 1:**
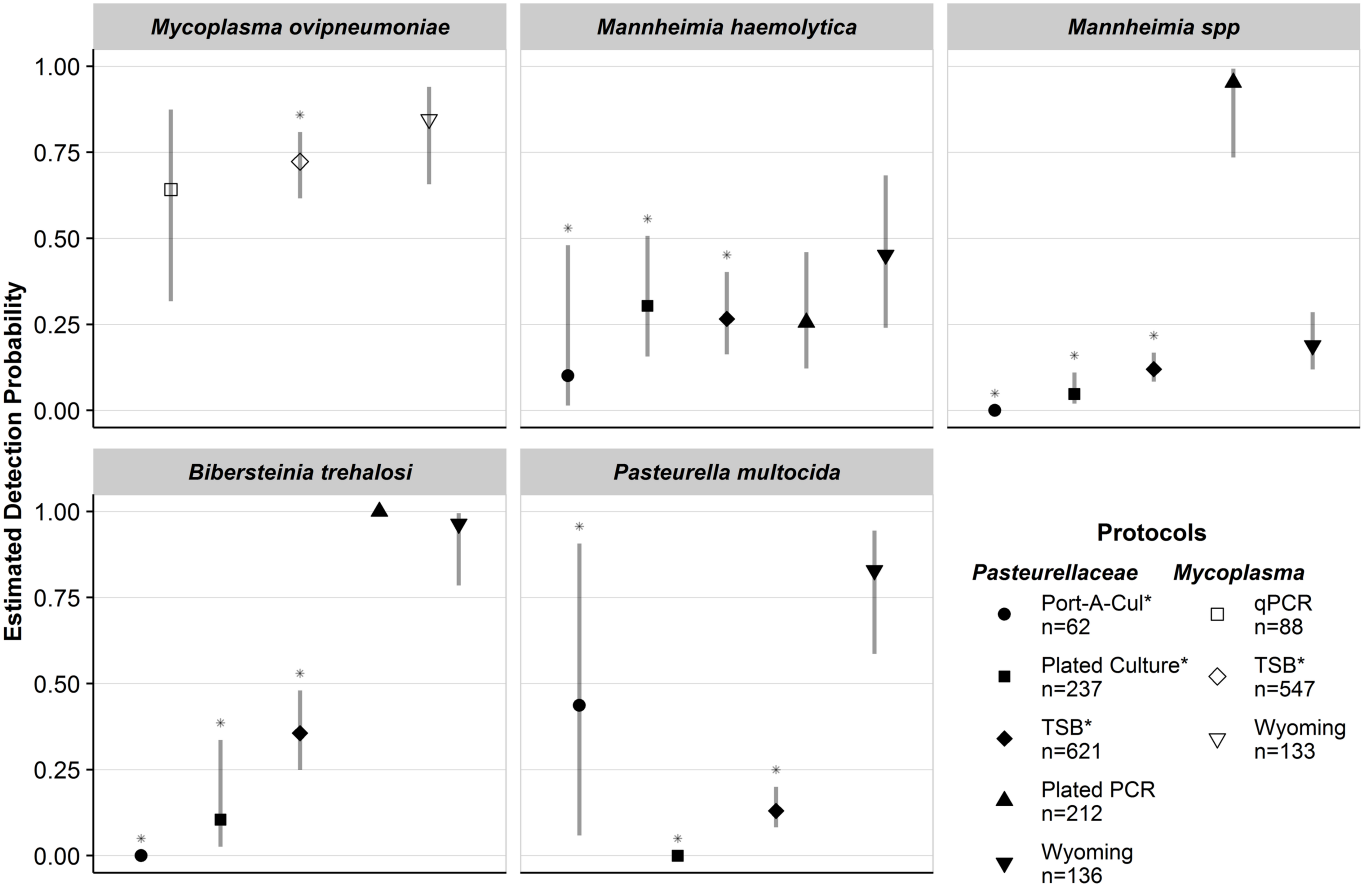
Estimated detection probabilities and 95% confidence intervals for five respiratory pathogens in bighorn sheep. One set of protocols was used to detect the four *Pasteurellaceae* organisms (shaded) and a separate set of protocols was used to detect *Mycoplasma ovipneumoniae* (not shaded). Detection probabilities for *Mannheimia haemolytica*, *Mannheimia spp*., and *Bibersteinia trehalosi* are for beta hemolytic or leukotoxigenic strains. Protocols that used fee-for-service diagnostic tests are indicated with an asterisk (*) in the legend and above the upper confidence limit. The total number of samples assessed using each protocol is indicated in the legend.

Detection probability for *M*. *ovipneumoniae* was greater than 0.60 for all diagnostic protocols that were evaluated and detection probabilities of all FFS diagnostic protocols (i.e., protocols that entailed shipping samples to WADDL for diagnostic testing) for *Pasteurellaceae* pathogens were less than 0.50 (Fig 1). No single diagnostic protocol used to detect the *Pasteurellaceae* pathogens resulted in estimated detection probabilities greater than 0.50 for all four targeted *Pasteurellaceae* pathogens. The FFS *Pasteurellaceae* protocols generally detected the targeted pathogens less effectively than the non-FFS diagnostic protocols: $\text{range }{\hat{p}}_{FFS}:\;0.0-0.44\;,\text{ range }{\hat{p}}_{non-FFS}:\;0.19-0.96$).

#### Mycoplasma ovipneumoniae

The estimated detection probability of *M*. *ovipneumoniae* using the Wyoming protocol was highest ($\hat{p}=0.85$, 95% CI: 0.66–0.94), however, the TSB protocol was comparable ($\hat{p}=0.72$, 95% CI: 0.62–0.81). Detection probability using the qPCR protocol was also comparable and estimated at 0.64 (95% CI: 0.32–0.87).

#### Mannheimia haemolytica

All protocols were relatively poor at detecting *M*. *haemolytica*, with estimated detection probabilities ranging from 0.10 (95% CI: 0.01–0.48) to 0.45 (95% CI: 0.24–0.68). Among the FFS protocols used to detect *M*. *haemolytica*, the Plated Culture and TSB protocols were comparable and performed best, with estimated detection probabilities of 0.30 (95% CI: 0.16–0.51) and 0.27 (95% CI: 0.16–0.40), respectively. Among non-FFS protocols, The Wyoming protocol had the highest estimated detection probability for *M*. *haemolytica* ($\hat{p}=0.45$, 95% CI: 0.24–0.68).

#### Mannheimia species

The Plated PCR protocol had the highest estimated detection probability for *Mannheimia* spp. ($\hat{p}=0.95$, 95% CI: 0.74–0.99). All other protocols were poor at detecting *Mannheimia* spp. ($\hat{p}<0.19$), with the TSB protocol having the highest estimated detection probability among the FFS protocols ($\hat{p}=0.12$, 95% CI: 0.08–0.16).

#### Bibersteinia trehalosi

The Wyoming protocol was good at detecting *B*. *trehalosi*, with an estimated probability of 0.96 (95% CI: 0.79–0.99). The detection probability for the Plated PCR protocol was estimated to be 1; however, this estimate is unreliable because *B*. *trehalosi* was only detected in two animals (out of 211) where the Plated PCR protocol was conducted. The highest estimated detection probability among protocols that used FFS diagnostic protocols for *B*. *trehalosi* was 0.36 (95% CI: 0.25–0.48) and was achieved using the TSB Protocol.

#### Pasteurella multocida

The highest detection probability for *P*. *multocida* was realized using the Wyoming protocol ($\hat{p}=0.83$, 95% CI: 0.59–0.94). Among FFS protocols, the estimated detection probability was highest for the Port-A-Cul protocol, 0.43, however precision of this estimate was poor (95% CI: 0.06–0.91). Estimated detection probabilities for *P*. *multocida* using the TSB and Plated Culture protocols were 0.13 (95% CI: 0.08–0.20) and 0 (inestimable 95% CI), respectively.

#### Inter-state differences in detection probability estimates

The independent analysis of two subsets of data collected in different states indicated that estimates of detection probability were similar for nearly all pathogen-protocol combinations that were investigated, (μ _difference_ = 0.04) and 95% confidence intervals overlapped substantially (S3 Appendix, Fig. S3.3). However, detection probability for *Mannheimia spp*. using the TSB Protocol was estimated at 0.01 (95% CI: 0.00–0.06) for the Montana subset, and was estimated at 0.31 (95% CI: 0.21–0.43) for the Wyoming subset. The differences between these estimates and the corresponding estimate obtained from the complete dataset were 0.11 (Montana subset) and 0.19 (Wyoming subset).

### Pathogen prevalence

**Fig 2:**
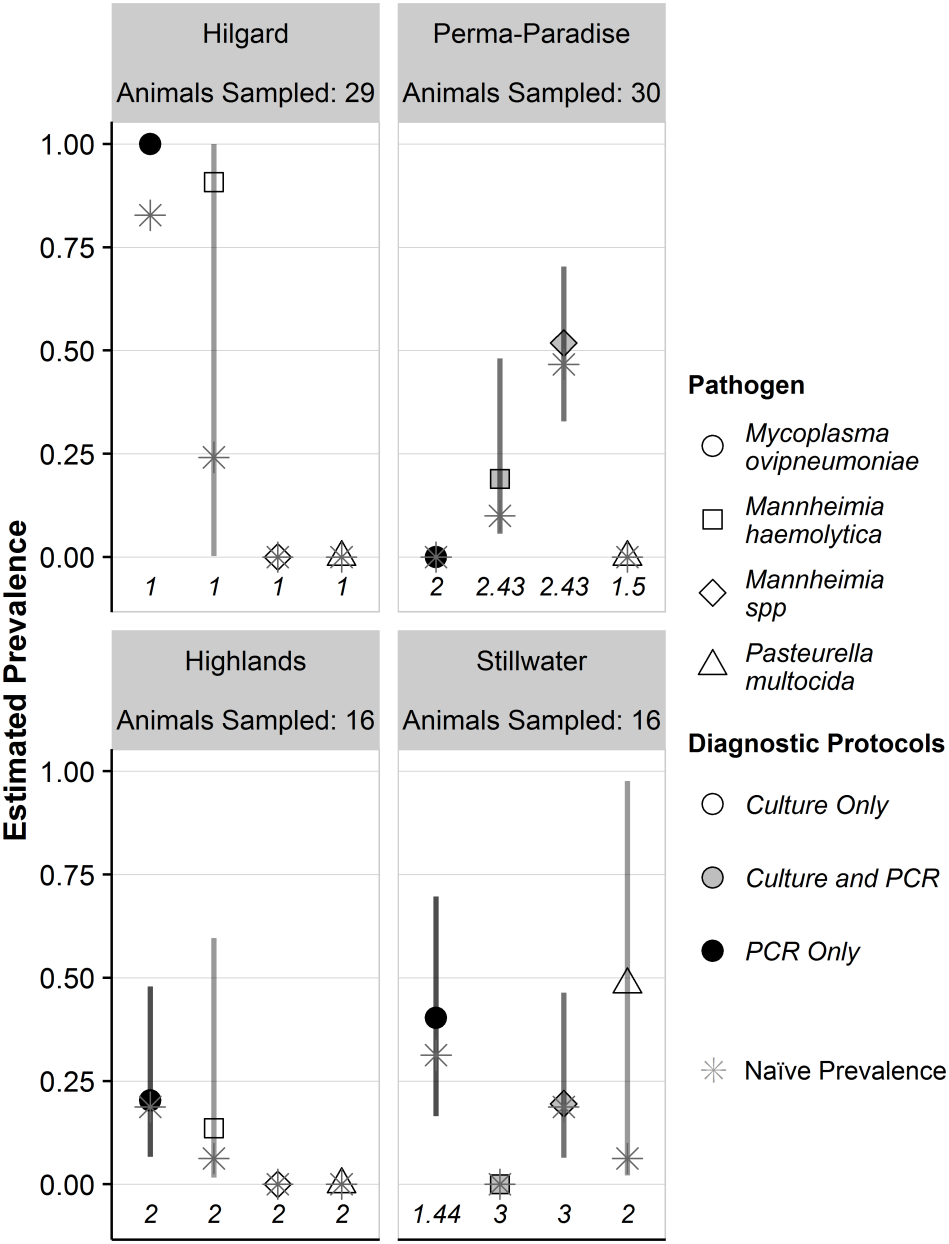
Estimated prevalence and 95% confidence intervals for four respiratory pathogens in four bighorn sheep populations. Prevalence estimates obtained where protocols only used culture tests to detect pathogens are indicated by open symbols, those obtained where protocols used a combination of culture and PCR tests are shown as gray symbols and those obtained where only PCR tests were used are shown by black symbols. The mean numbers of protocols conducted per animal are shown across the x-axis of each panel. Naïve prevalence estimates (the proportion of animals a pathogen was detected in for a given sampling occasion) are indicated with gray asterisks. Prevalence estimates for *Mannheimia haemolytica* or *Mannheimia spp*. are for beta hemolytic or leukotoxigenic strains. 95% confidence intervals were inestimable when detection probability was estimated at 0 or 1.

We present prevalence estimates and associated 95% confidence intervals from four different sampling occasions (sampling of a specific population over a winter season) that best represent a range of reasonable sampling intensities; these sampling occasions include the Hilgard population in 2013/2014, the Perma-Paradise population in 2014/2015, the Highlands population in 2015/2016, and the Stillwater population in 2014/2015. The number of animals from each population that were sampled using each diagnostic protocol is shown in S3 Appendix. Among these populations, pathogen prevalence estimates ranged from 0 (not detected in the sampling occasion) to 1. Excluding these extremes, estimates of pathogen prevalence ranged from 0.14 to 0.91 (Fig 2).

When only FFS diagnostic protocols were used (i.e., no PCR tests were conducted) to detect *Pasteurellaceae* pathogens (also corresponding to conducting a maximum of two diagnostic protocols per animal), precision of prevalence estimates was poor, with 95% confidence intervals including over 50% of all possible prevalence values (i.e., parameter space). Precision of prevalence estimates was higher when two or more diagnostic protocols were conducted per animal and at least one of the protocols included a PCR test; however 95% confidence intervals still always included at least 37% of all possible prevalence values (Fig 2). Naïve prevalence estimates were generally similar to prevalence estimates that accounted for detection probability when multiple protocols (either replicating a single protocol or conducting different protocols) were conducted per animal (Fig 2).

#### Mycoplasma ovipneumoniae

Among the sampling occasions where *M*. *ovipneumoniae* was detected, the differences between the naïve estimates of prevalence for *M*. *ovipneumoniae* and the estimates that accounted for detection probability were small relative to those differences for other pathogens: 0.17 when a single diagnostic protocol was conducted from each of 29 animals using the TSB protocol (Hilgard), 0.09 when an average of 1.4 diagnostic protocols were conducted per each of 16 animals using the TSB and qPCR protocols (Stillwater), and 0.01 when two diagnostic protocols were conducted per each of 16 animals by replicating the TSB protocol (Highlands; Fig 2). The corresponding 95% confidence interval for the prevalence estimate that accounted for detection probability included 53% (0.17–0.70) and 41% (0.07–0.48) of all possible prevalence values for the Stillwater and Highlands populations, respectively. The prevalence estimate for the Hilgard populations was at a parameter boundary (estimated to be 1) and the 95% confidence interval was inestimable.

#### Mannheimia haemolytica

Among the sampling occasions where *M*. *haemolytica* was detected, the naïve prevalence estimate for *M*. *haemolytica* and the prevalence estimate that accounted for detection probability were dissimilar (Δ = 0.67) when a single diagnostic protocol was conducted per each of 29 animals using the TSB protocol (Hilgard), but less so (Δ = 0.07 and Δ = 0.09) when at least two diagnostic protocols were conducted per animal in the Highlands (TSB protocol conducted twice per each of 16 animals) and Perma-Paradise (average of 2.4 diagnostic protocols conducted per each of 29 animals) populations. The corresponding 95% confidence intervals for the prevalence estimates that accounted for detection probability were wide, including 99% (0.01–1), 58% (0.02–0.60), and 42% (0.06–0.48) of all possible prevalence values for the Hilgard, Highlands, and Perma-Paradise populations, respectively.

#### Mannheimia species

*Mannheimia* spp. was detected in the Stillwater and Perma-Paradise sampling occasions, (16 and 30 animals sampled, respectively; Fig 2). The estimates of naïve prevalence for the Stillwater and Perma-Paradise populations were both very similar to the estimates that accounted for detection probability (Δ = 0.01 and Δ = 0.05, respectively). However, the 95% confidence intervals for the estimate of prevalence that accounted for detection probability were imprecise, including 40% (0.06–0.46) and 37% (0.33–0.70) of all possible prevalence values for the Stillwater and Perma-Paradise populations, respectively.

#### Pasteurella multocida

*P*. *multocida* was only detected in the Stillwater population, where its presence was assessed by conducting two diagnostic protocols per each of 16 animals (Fig 2). There was a large difference between the naïve prevalence estimate and the prevalence estimate that accounted for detection probability (Δ = 0.42). The 95% confidence interval for the prevalence estimate that accounted for detection probability included 96% of all possible prevalence values (0.02–0.98).

### Power to detect pathogens at the population-level

#### Differences among pathogens using TSB protocol

**Fig 3:**
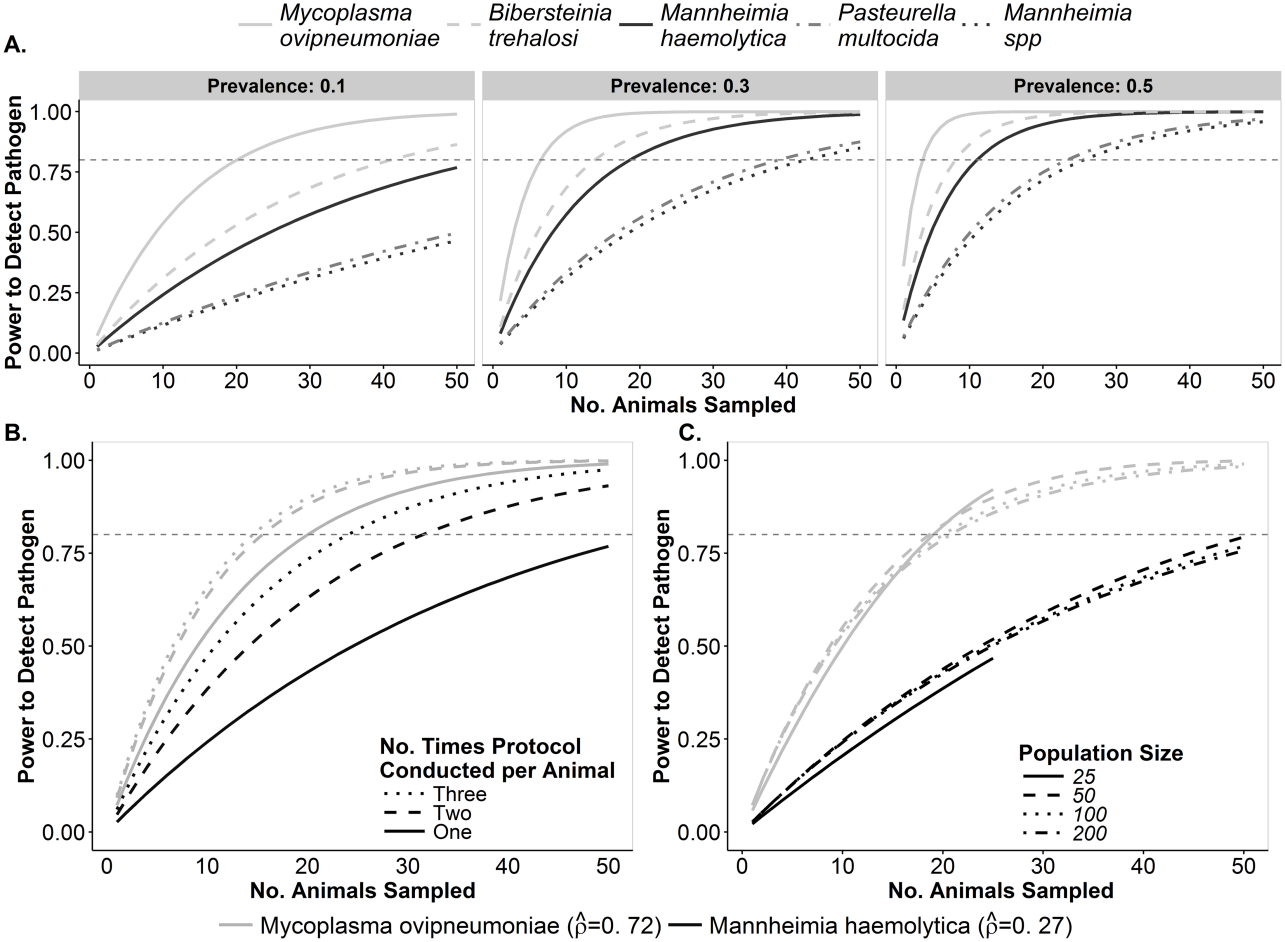
Power to detect five respiratory pathogens in bighorn sheep populations using the TSB protocols. Variability in power to detect pathogens at the population level is shown as it relates to different pathogens, population size, pathogen prevalence, number of animals sampled, and number of times protocols are conducted per animal. For all panels, each curve illustrates the power to detect each pathogen (y-axis) given the number of animals sampled from a population (x-axis). The horizontal dashed-gray line across each panel represents adequate (i.e., 80%) detection power. A. Variability in the power to detect each pathogen when the TSB protocol is conducted once per animal at three levels of pathogen prevalence in a population of 100. B. Effects of conducting TSB protocol multiple times per animal on power to detect two pathogens with either relatively high or relatively low detection probability in a population of 100 animals with 10% pathogen prevalence. C. Effect of population size on power to detect two pathogens with either relatively high or relatively low detection probability in a population of 100 animals with 10% pathogen prevalence.

Power to detect the respiratory pathogens using the TSB protocol varied greatly, and the pathogens clustered into three groups. Power to detect *M*. *ovipneumoniae* was greatest, followed by *B*. *trehalosi* and *M*. *haemolytica*, then by *Mannheimia spp*. and *P*. *multocida* (Fig 3A). Under the default conditions (i.e., diagnostic protocol: TSB, animals sampled: 25, number of times protocol was conducted per animal: 1, pathogen prevalence: 0.10, population size: 100), power to detect *M*. *ovipneumoniae* was estimated at 0.87, *B*. *trehalosi* 0.61; *M*. *haemolytica* 0.51; *P*. *multocida* 0.29, and *Mannheimia spp*. 0.27. Increasing the number of animals sampled from 25 to 50 led to achieving adequate power (>0.80) to detect *B*. *trehalosi*, but no other targeted *Pasteurellaceae* pathogen (Fig 3A).

#### Differences among diagnostic protocols

**Fig 4:**
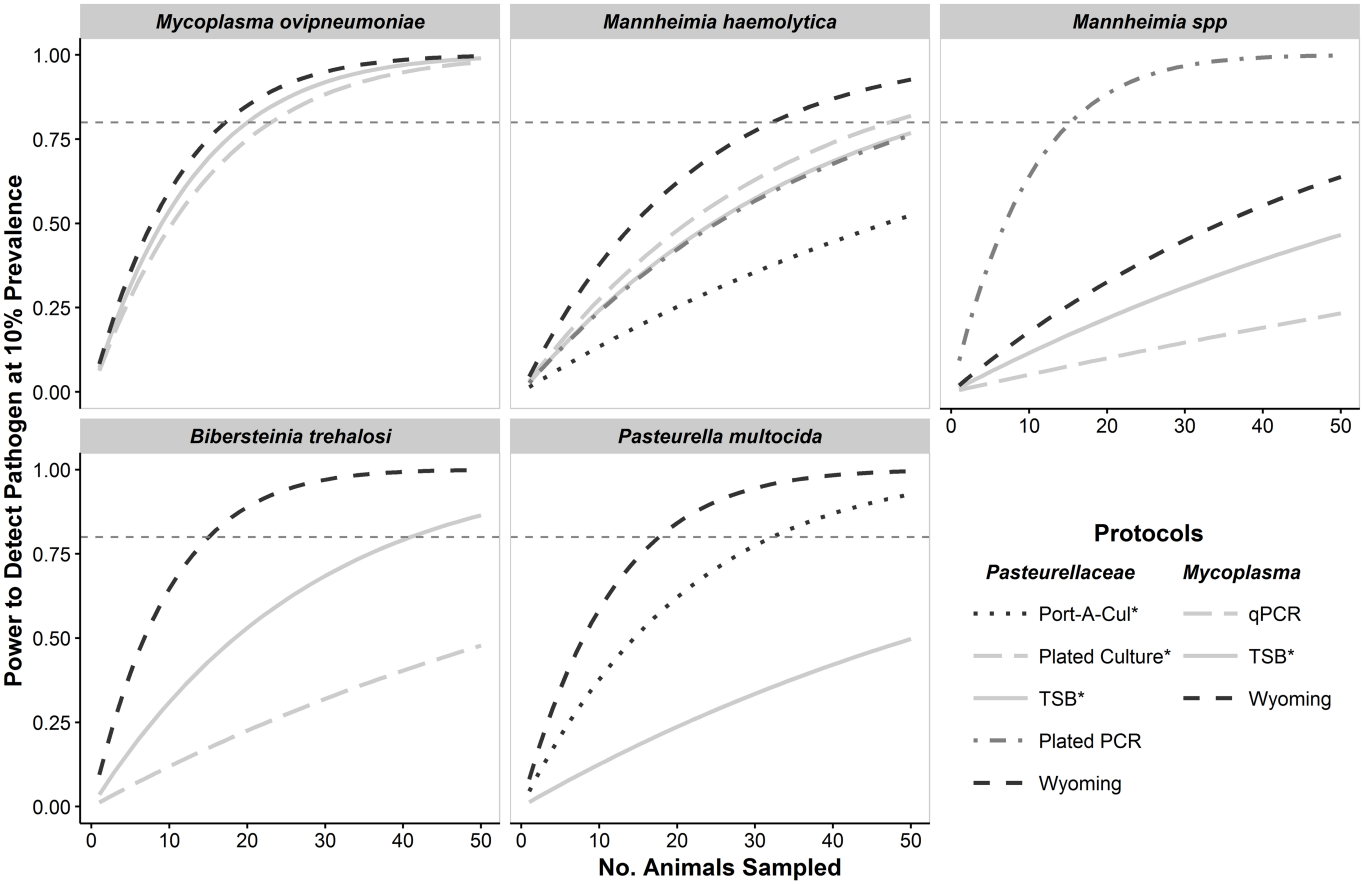
Power to detect five respiratory pathogens in bighorn sheep populations using different diagnostic protocols. For all panels, each curve illustrates the power to detect each pathogen at the population-level (y-axis) given the number of animals sampled from a population (x-axis) when the specified protocol was conducted once per animal. Within each panel, protocols where detection probability was estimated at zero or one are not displayed. The horizontal dashed-gray line across each panel represents adequate (i.e., 80%) detection power.

Diagnostic protocol affected the power to detect each pathogen and there was a strong difference between the power of the TSB protocol and the protocol with the most power to detect each pathogen (Fig 4). For example, under default conditions, the power to detect *B*. *trehalosi* and *P*. *multocida* using the TSB protocol was estimated to be 0.61 and 0.29, respectively. In contrast, the power to detect these pathogens under default conditions using the Wyoming protocol was estimated to be 0.94 and 0.91, respectively. Although the difference in detection probability between the TSB and the most powerful protocols for *M*. *haemolytica* was not as substantial as for the other *Pasteurellaceae* pathogens, there were still notable differences in detection power. Under default conditions, the estimated detection power using the TSB protocol was 0.51 compared to 0.71 using the Wyoming protocol. There was little difference in the power to detect *M*. *ovipneumoniae* between the TSB protocol and the Wyoming protocol and every protocol provided adequate power to detect *M*. *ovipneumoniae* when the protocols were conducted once per each of 25 animals (Fig 4). Every *Pasteurellaceae* pathogen, except *Mannheimia* spp. could be reliably detected by conducting the Wyoming protocol once on each of 35 animals (Fig 4).

#### Sampling effort

Increasing the number of animals sampled improved power to detect pathogens, but rates of improvement declined as the number of animals sampled increased (Fig 3A). For example, increasing the number of animals sampled for presence of *M*. *haemolytica* (under default conditions) from 15 to 25 increased the estimated detection power from 0.34 to 0.51 (Δ = 0.17). Sampling 35 animals under default conditions increased the estimated detection power to 0.63 (Δ = 0.12). When 15 animals were sampled under default conditions, there was not adequate detection power for any pathogen and the estimated power to detect each of the *Pasteurellaceae* pathogens was less than 0.50. When 35 animals were sampled under otherwise default conditions, adequate power was achieved for *M*. *ovipneumoniae*, but not for any of the *Pasteurellaceae* pathogens (Fig 3A).

**Fig 5:**
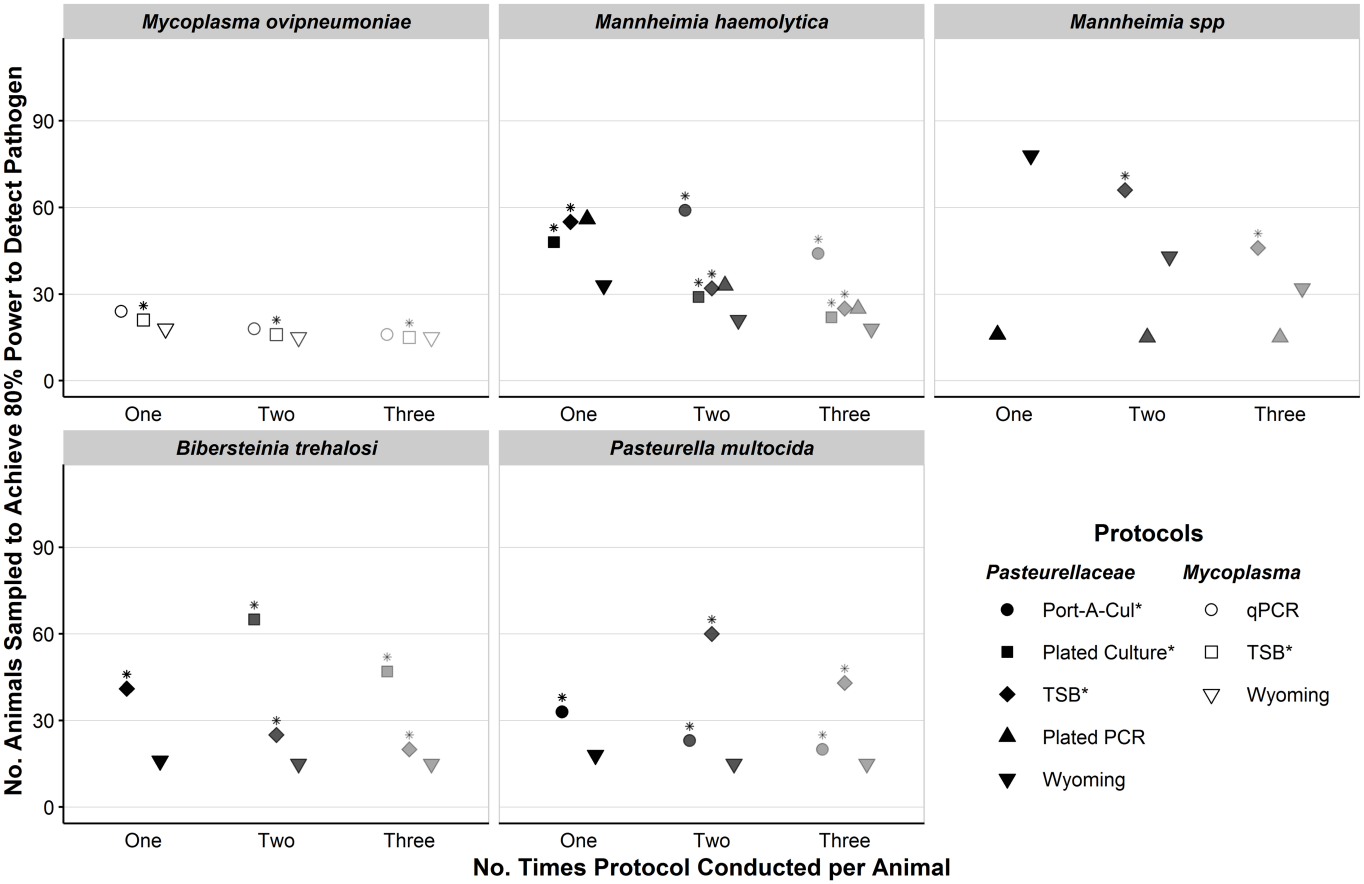
Number of bighorn sheep to sample to achieve adequate detection power to detect respiratory pathogens. Minimum numbers to sample are those estimated to provide 80% power to detect a pathogen at 10% prevalence in a population of 100 animals using the specified protocol and number of samples per animal. One set of protocols was used to detect Pasteurellaceae organisms (shaded) and a separate set of protocols was used to detect *Mycoplasma ovipneumoniae* (not shaded). Protocols that used fee-for-service diagnostic tests are indicated with an asterisk (*) in the legend and above their symbols. Within each panel, protocols where detection probability was estimated at zero or one are not displayed. The same is true for protocols where the estimated number of animals required to sample in order to attain adequate detection power was greater than 100.

Detection power was markedly improved by conducting protocols multiple times per animal when detection probabilities were low. The largest gains occurred between conducting protocols one and two times per animal, and there were diminishing returns from conducting protocols a third time (Fig 3B). Under the default conditions described above where 25 animals were sampled, conducting the TSB protocol twice per animal increased estimated detection power of *M*. *haemolytica* from 0.51 to 0.72 (Δ = 0.21) and conducting the TSB protocol three times per animal further increased estimated detection power to 0.81 (Δ = 0.09). In contrast, power to detect *M*. *ovipneumoniae* under default conditions improved to 0.87, 0.94, and 0.95 when the TSB protocol was conducted one, two, and three times per animal, respectively. When 35 animals were sampled and the TSB protocol was conducted multiple times per animal using, adequate detection power was also achieved for *M*. *haemolytica* (conducting protocol twice per animal), *B*. *trehalosi* (conducting protocol twice per animal), but not for *Mannheimia* spp. or *P*. *multocida* (Fig 5).

#### Pathogen prevalence

Pathogen prevalence had a strong influence on the number of animals that would need to be sampled to achieve adequate detection power. For example, adequate power (≥0.80) to detect *M*. *haemolytica* in a population at a prevalence of 0.50 could be attained by sampling just 11 animals under default conditions (Fig 3A). However, with a lower prevalence of 0.10, 55 animals would need to be sampled to attain adequate detection power (Fig 5). The effect of pathogen prevalence on necessary sampling effort was non-linear and most pronounced when prevalence was low. An increase in pathogen prevalence from 0.10 to 0.30 decreased the number of animals to be sampled to attain adequate power from 55 to 19 (Δ = 36 animals). However, a further increase in prevalence from 0.30 to 0.50 only decreased the minimum number of animals to sample to 11 (Δ = 8 animals, Fig 3A).

#### Population size

Power to detect pathogens was minimally affected by population size (Fig 3C). Under default conditions, the estimated detection power for *M*. *haemolytica* was 0.47 in a population of 25 compared to 0.51 in a population of 100. Under default conditions for *M*. *ovipneumoniae*, detection power was 0.92 in a population of 25 and 0.87 in a population of 100. Small population size had the most notable effect on the power to detect pathogens simply due to the limited number of animals that could possibly be sampled (Fig 3C).

## Discussion

Our findings indicate that live-sampling of bighorn sheep for respiratory pathogens using diagnostic protocols that are readily available to most wildlife management agencies (i.e., available through an FFS laboratory) can lead to biased assessments of respiratory pathogen communities. While the diagnostic test to detect *M*. *ovipneumoniae* offered by the FFS laboratory used in this study (WADDL) uses PCR with a high detection probability, only culture tests are offered by FFS laboratories to detect and identify *Pasteurellaceae* pathogens in bighorn sheep. Diagnostic protocols that relied solely on an FFS culture test for detection had low estimated detection probabilities (<0.50) for all *Pasteurellaceae* pathogens that were assessed. Low detection probability of these protocols may be due in large part to diminished viability of targeted organisms during the process of delivery to the laboratory rather than sensitivity of the diagnostic test itself [17,30]. A recent study comparing test results from numerous diagnostic laboratories, including the two used in this study, found generally high levels of agreement among laboratories in detecting bighorn sheep respiratory pathogens in lung-homogenate samples derived from a common sample, further suggesting that the primary source of detection error occurs prior to when laboratories receive samples [31]. This reiterates the importance of careful and mindful sample collection and handling, particularly when samples are tested for pathogens using culture tests.

Low detection probability of *Pasteurellaceae* pathogens using FFS protocols makes simple assessment of species presence at the population-level unreliable when species are at low prevalence and populations are not intensively sampled. These conclusions generally corroborate those of the previous investigation of detection probability of *Pasteurellaceae* pathogens [15]. Although our specific findings apply to live-sampling bighorn sheep by swabbing the nasal cavity or tonsillar crypts, different findings between culture and PCR diagnostic tests applied to the same lung tissue suggests that detection error also affects assessment of pathogen communities in lung tissue [8]. Thus, an assessment of detection probability applied to the sampling of lung tissues is warranted.

Naïve prevalence estimates of *Pasteurellaceae* pathogens are strongly biased when FFS diagnostic protocols are used, unless protocols are conducted multiple times per animal. Given poor detection power and biased naïve prevalence estimates, any true associations between the presence of *Pasteurellaceae* organisms and historic or current respiratory disease in bighorn sheep would likely be unobservable using these protocols. The specificity issues that led to our generalized classification system for *Mannheimia* genus organisms further limit the ability to understand what role *Pasteurellaceae* pathogens play in bighorn sheep respiratory disease. In contrast to the *Pasteurellaceae* pathogens, high detection probability for *M*. *ovipneumoniae* likely leads to more consistent detection and less biased naïve prevalence estimates in bighorn sheep populations where it is hosted.

We found that prevalence of any pathogen is estimated with poor precision unless intensive sampling is employed (i.e., many animals are sampled and protocols are conducted multiple times per animal), matching general expectations when detection error occurs [32]. Although *M*. *ovipneumoniae* could be reliably detected in a population by conducting a single protocol on a modest number of animals, its prevalence was estimated with low precision unless more sampling effort was invested. Therefore, variability in observed pathogen prevalence among different populations or different years within a population could be explained by either sampling variation or true variation in prevalence. Without accounting for differences in detection probability and sampling effort, differences in true prevalence remain unknown.

A simple and relatively inexpensive measure that wildlife management agencies can take to improve their ability to accurately characterize respiratory pathogen communities is to collect and assess two or three tonsil swabs from each live-sampled animal for *Pasteurellaceae* pathogens using FFS diagnostic protocols. Conducting protocols multiple times per animal would also provide agencies the ability to assess detection probability of their specific diagnostic protocols. Our results suggest that 30 to 35 animals need to be sampled from a bighorn sheep population to reliably assess (>80% power) presence of most *Pasteurellaceae* pathogens and *M*. *ovipneumoniae*. Either sampling 35 animals and conducting the *Pasteurellaceae* TSB protocoltwice per animal or sampling 30 animals and conducting the *Pasteurellaceae* TSB protocol three times per animal is predicted to result in adequate power to detect *M*. *haemolytica* and leukotoxigenic *B*. *trehalosi* at 10% prevalence. In either of these scenarios, *M*. *ovipneumoniae* can be reliably detected by conducting the TSB protocol just once per animal. These recommendations are based on a population size of 100 animals; the recommended level of sampling will produce slightly lower detection power for larger populations and greater detection power for smaller populations.

At least one protocol was estimated to have high detection probability for most *Pasteurellaceae* pathogens; however, detection probability for *M*. *haemolytica* was not substantially improved with any protocol. Our results predict that 32 animals must be sampled (in a population of 100 animals) using the most powerful protocol (Wyoming Protocol) to achieve adequate detection power for *Mannheimia haemolytica*. The limited improvement in estimated detection probability for *M*. *haemolytica* using the Wyoming protocol compared to the TSB or Plated PCR protocols corresponds closely to what would be expected by collecting two swabs, suggesting that the improvement may simply be the result of collecting two samples per animal. The different detection probabilities for leukotoxigenic *Mannheimia spp*. pathogens detected in different states (using the TSB protocol) may be explained by animals sampled in the two states hosting different species within the *Mannheimia* genus. This assertion is supported by the observation that in winter 2015/2016 (the only year when *Mannheimia ruminalis* could be identified) most *Mannheimia spp*. isolates (as defined in our analysis) from Montana were identified by WADDL as *Mannheimia ruminalis* while most isolates from Wyoming were identified by WADDL as unidentified *Mannheimia species*. I The detection power estimates we provide for *Mannheimia spp*. using the TSB protocol represent a compromise between two disparate estimates we obtained for samples collected in different state; additional investigation using diagnostic tests with improved specificity is needed to provide better sampling recommendations for this class of pathogens. Lastly, there is some evidence to suggest that *P*. *multocida* may be more readily detected in nasal swabs than tonsil swabs as a higher percentage of nasal swabs in our dataset that were assessed using the *Pasteurellaceae* TSB protocol tested positive for this pathogen.

Reliable detection of pathogens at the population-level, as defined in this study, still results in a 20% chance of a Type II error (false negative) for each pathogen. Assuming pathogen species are independently distributed in a population and independently detected, thee power to simultaneously detect multiple pathogen species in a population is further reduced to the product of the power to detect each individually. Additionally, technological improvements have enabled increased specification of some species to the strain-level; for example recent work has identified multiple strains of *M*. *ovipneumoniae* circulating in bighorn sheep populations [33]. If it is assumed that different strains of a pathogen species share a common detection probability, the power to simultaneously detect multiple strains of pathogen species is also reduced to the product of the power to detect each strain individually. Thus, the full set of respiratory pathogens hosted by free-ranging bighorn sheep populations will likely never be characterized with certainty. However, quantification of this uncertainty is possible using the concepts presented here and would lead to more accurate inference and informed management decisions.

One limitation of this study is the unbalanced application of the diagnostic protocols across study populations. Accordingly, our estimates of detection probability may not be entirely generalizable to other study populations or research teams. Additional testing of these protocols would be valuable to validate the predictive ability of our estimates. Nevertheless, increased awareness of detection error in disease sampling should lead to improved understanding of disease processes dynamics. These concepts can be applied to other diagnostic protocols (for which detection probability has been estimated) by using free and user-friendly online software (http://epitools.ausvet.com.au [34]), and manually calculating the per-animal detection rate if protocols are conducted multiple times per animal. We plan to develop additional user-friendly tools that build upon these concepts and will aim to help wildlife managers and researchers better interpret previous disease testing and plan future disease sampling.

Poor detection power associated with FFS diagnostic protocols combined with hundreds of bighorn sheep translocations across North America suggest it is probable that *Pasteurellaceae* have been unknowingly introduced to new regions and populations. Suspected poor detection probability for *M*. *ovipneumoniae* prior to development of FFS PCR and serology tests [35], likely also resulted in the unknown introduction of this pathogen to new regions or host populations. Typically, bighorn sheep populations chosen to be source populations for translocations are those experiencing population growth, and thus, not exhibiting noticeable symptoms of respiratory disease. However, such populations may still host respiratory pathogens capable of causing disease [5]. We recommend that bighorn sheep source populations be thoroughly sampled for respiratory pathogens using appropriate diagnostic protocols and sampling intensities and that uncertainty associated with not detecting pathogens be accounted for in order to determine the extent to which these respiratory pathogens are present.

Resident respiratory pathogens within bighorn sheep populations exhibiting satisfactory demographic performance may pose a risk of future all-age respiratory disease epizootics within those populations [5]. Given poor power to detect respiratory pathogens at low prevalence using FFS live-sampling diagnostic protocols, initial detection of pathogens in bighorn sheep populations following observed respiratory disease may reflect an increase in prevalence or detectability [32,36] rather than an introduction of the pathogen to the population. Thus, taking measures to rigorously assess pathogen presence in populations with and without obvious signs of respiratory disease can provide multiple benefits for bighorn sheep conservation including informing translocation decisions, providing evidence for different sources of respiratory disease epizootics (i.e., novel vs resident pathogens) and elucidating additional measures that may be possible to prevent or mitigate respiratory disease in bighorn sheep.

## Conclusions

Rigorous assessment of bighorn sheep respiratory pathogen communities is important for wildlife management agencies to inform potential translocations and to contribute to our understanding of respiratory disease in bighorn sheep. If adequate detection power for respiratory pathogens is not regularly achieved when bighorn sheep populations are sampled, test results have limited utility and are potentially misleading. Our findings suggest that it is inadvisable to make strong inferences regarding causative agents of respiratory disease based on comparative estimates of pathogen prevalence unless resources are invested to accurately characterize pathogen communities. Relative to the total cost of sampling bighorn sheep, additional expenses to improve characterization of respiratory pathogen communities are rather modest. Improved and consistent characterization of respiratory pathogen communities across numerous populations will help clarify the predominant causes of respiratory disease epizootics. The roles of the suspected respiratory pathogens, the predominant proximate causes of respiratory disease epizootics, and factors that could promote bighorn sheep populations to maintain vigor despite presence of respiratory pathogens are topics that may require multi-agency coordination and focused efforts to address. The information generated by this study may be used to inform future respiratory pathogen sampling efforts and allow rigorous comparison of pathogen communities assessed by different agencies and diagnostic protocols.

## Supporting information

S1 Appendix Occupancy modeling assumptions. (DOCX)

S2 Appendix Detailed descriptions of diagnostic protocols. (DOCX)

S3 Appendix Assessing inter-state differences in detection probability estimates. (DOCX)

S4 Appendix Derivation of detection power solution. (DOCX)

S5 Appendix Detection power curves for all pathogen-protocol combinations. (DOCX)
