# Supplementary material for: Assessing respiratory pathogen communities in bighorn sheep populations: Sampling realities, challenges, and improvements
Source: PLoS One. 2017 Jul 14;12(7):e0180689. doi: 10.1371/journal.pone.0180689 (PMC5510838; doi:10.1371/journal.pone.0180689)
Supplement: S1 Appendix — (DOCX) [file pone.0180689.s001.docx]

**S1 Appendix. Fulfilment of Occupancy Modeling Assumptions**

Occupancy modeling has recently been recognized as a framework well-suited for evaluating detection probability and prevalence of pathogens in wildlife populations [1–4]. However, there has been relatively little discussion of several of the assumptions associated with occupancy models in the application to pathogen testing in wildlife populations. As such, we present the fundamental assumptions of these models, general factors that may lead to violation of these models’ assumptions, and how our application met or potentially did not meet the assumptions.

Assumptions of this modeling approach include: (1) the infection status of an animal does not change during the sampling period; (2) all animals have the same probability of the pathogen being present or heterogeneity is accounted for; (3) the probability of a positive detection from a sample is the same for all animals that host the pathogen or heterogeneity is accounted for; (4) detections are independent; and (5) there are no false positive detections [1]. Each assumption is discussed in detail below:

1. *The infection status of an animal does not change during the sampling period.*

Typically, multiple samples are collected from animals within a very short period of time, which suggests that this assumption is likely met in most pathogen sampling situations. A possibility that should be considered, however, is whether collection of samples from the same site reduces the density of the target pathogens remaining for subsequent samples to come into contact with. This occurrence would lead to low-biased estimates of detection probability if left unaccounted for.

We assessed evidence for whether swab samples denuded the sample site of target pathogens by systematically assigning protocol order and recording order of collection for swabs collected from a subset of animals sampled in Montana (n=106). We then assessed evidence for a relationship between detection probability and swab order , after accounting for the protocol, for each pathogen by censoring the datasets to only include samples from animals where a particular pathogen was detected and then fitting a single-season, single species occupancy model [5] to the resulting datasets. The resulting datasets consisted of 55 tests from ten animals for *Mannheimia haemolytica,* 180 tests from 37 animals for (unclassified leukotoxigenic) *Mannheimia species,* and 16 tests from eight animals for *Mycoplasma ovipneumoniae.* There were insufficient detections for leukotoxigenic *B. trehalosi* (n=1) and *P. multocida* (n=2) to conduct this analysis. After accounting for protocol, there was no evidence for a relationship between detection probability and swab order for *Mannheimia haemolytica* (β_order_ = -0.21, 95% CI: -0.73 to 0.30), *Mannheimia species (*β_order_ = 0.174, 95% CI: -0.73 to 0.30), or *Mycoplasma ovipneumoniae (*β_order_ = 0.4, 95% CI: -1.92 to 1.05), as the 95% confidence intervals for the swab order “effect” all substantially overlapped zero. These results suggest that the assumption of closure was not violated in this study.

1. *All animals have the same probability of the pathogen being present, or heterogeneity is accounted for.*

An obvious way by which this assumption could be violated in a multi-population study, such as this, would be failure to distinguish which population animals were sampled from, as the probability of pathogen presence (i.e., prevalence) varies among populations. Our modeling approach allowed pathogen prevalence to vary for each population-year (i.e., differences in pathogen prevalence among bighorn sheep populations and differences among years within populations were accounted for), giving us confidence that we adequately accounted for heterogeneity in pathogen presences at the population level. It is possible that probability of pathogen presence varies among individuals within population-years. Factors potentially leading to this variation could include temporal variation (within a season), sub-population structure, or age and sex. It is unlikely that these factors had substantial impacts on our results as our sampling targeted adult females on primary winter ranges for each population: 417 of 479 (87%) animals sampled in this study were adult (>18 months old) females. Accordingly, our results are most applicable to the sampling of adult female bighorn sheep. Issues related to within-season variation in pathogen prevalence can be avoided by sampling animals in a concentrated time-period. For 24 of 34 population-years in our dataset, all animals were sampled within 3 days of each other and for 28 of 34 population-years all animals were sampled within 34 days of each other. Although animals in six population-years were sampled over a period of two to three months (December-March), these animals only comprised 65 of the 479 (14%) animals sampled in this study.

1. *The probability of a positive detection from a sample is the same for all animals that host the pathogen, or heterogeneity is accounted for.*

One obvious potential cause of heterogeneity in probability of pathogen detection among animals is variability in pathogen load, which may in turn be associated with active infection or clinical symptoms. Heterogeneity in detection probability associated with variability in pathogen load could result in under-estimating detection probability in individuals with active infections and over-estimating detection probability in individuals with latent infections. Culture-independent quantitative PCR (qPCR) offers a potential method to quantify and address heterogeneity in pathogen load and previous studies have used qPCR threshold cycle (C_t_) values to index pathogen load of host organisms [2,3]. We caution, however, that qPCR C_t_ values are an index of pathogen load collected in the *sample* which may be affected by factors other than pathogen load in the host in certain systems. In general we did not have access to culture-independent qPCR for our sampling; an exception was the *Mycoplasma ovipneumoniae* qPCR protocol that was conducted on a subset of individuals. In order to provide inferences relevant to the of organizations who sample the most bighorn sheep, we focused on protocols which we felt were most-used or could be readily adopted by wildlife management agencies; as such we did not pursue developing additional culture-independent qPCR diagnostic protocols for this study and were unable to use this approach to index pathogen load. The majority of animals that we sampled did not show noticeable clinical signs of active respiratory infections (e.g. nasal discharge or coughing) upon sampling; we were unable to quantify the exact number as we did not systematically record whether clinical signs of respiratory infections were observed for each animal. However, our experience suggests that active respiratory infections may not be reliably observed during animal handling: One individual sampled as part of this study that showed no external signs of respiratory infection during the sampling process died shortly after capture and necropsy revealed active pneumonia. In summary, while heterogeneity in pathogen load may affect detection probability for respiratory pathogens in bighorn sheep, there may not currently be a feasible method to accurately measure pathogen load for a large enough sample of animals to gain strong inferences.

Another factor that could cause heterogeneity in detection probability estimates among individual animals is undistinguished strain-variation of pathogens. For example, previous investigation of detection probability for *Pasteurellaceae* in bighorn sheep reported some variability in detection probability estimates among biovariants within the *Pasteurellaceae* species that we investigated [4]. However, these findings were based on a limited sample size and considered both hemolytic and non-hemolytic biovariants within the *Mannheimia* and *Bibersteinia* genera, whereas this study only considered hemolytic or leukotoxigenic biovariants within these genera. Nevertheless, we suggest that poor ability to classify pathogen species within the *Mannheimia* genus may explain the heterogeneity in estimated detection probability that this study observed between organisms in the *Mannheimia* genus that were isolated in Montana and Wyoming (Appendix S3). It has also been documented that multiple strains of *Mycoplasma ovipneumoniae* can exist within and among bighorn sheep populations [6]. Although it is known that different strains of *Mycoplasma ovipneumoniae* and *Pasteurellaceae* exist within and among bighorn sheep populations, tests to distinguish strains within species are not readily-available and our study did not have the resources necessary to sample a sufficient number of animals and conduct the requisite diagnostic tests to assess strain-specific variation in detection probability. If heterogeneity in detection probability due to strain-type does exist, the detection probability estimates we provide will be biased low for more readily-detectable strains and biased high for more fastidious strains. Until specification of bighorn sheep respiratory pathogens into different strains is more feasible for wildlife agencies and researchers, these findings serve as the best available information to generate sampling recommendations for these pathogens.

1. *Detections are independent within and between animals*

Independence of data points is a ubiquitous assumption among statistical methods and violation of this assumption results in exaggerated precision in parameter estimates. The assumptions of within-animal and between-animal independence seem to be well-met in this application because the process of detecting the pathogens in samples was separate from the process of collecting the samples themselves, which were relabeled to ensure “blindness” when multiple samples from the same animal were sent to a laboratory. However, some dependence exists in this study between the Plated Culture and Plated PCR *Pasteurellaceae* protocol and the two protocols stemmed from the same swab sample collected from the animal (see Appendix S2). The consequence of this lack of independence depends on the amount of detection error for these protocols that occurs during the process of collecting the sample from the animal versus the process of delivering target organisms to the diagnostic laboratory. Another source of dependence in this study could be the shipment of samples to the laboratory in multiple batches. Batches of samples assessed using the same diagnostic protocols were all handled and shipped in the same manner to minimize this source of dependence; however the batches may have been treated unequally after being shipped.

1. *There are no false-positive detections.*

This assumption has received the most attention among studies that have adopted the occupancy modeling framework to investigate pathogen dynamics in wildlife populations, as the consequences of this misdiagnosis are severe and obvious. False-positive detections in diagnostic testing are typically considered in light of imperfect test specificity; however other potential sources include mislabeled samples or contamination between samples collected from different animals. We took extreme care to ensure the latter two sources of false-positive error did not occur. Although statistical approaches have been developed to account for false positives in this framework [1,7], the identification of false-positives through a “gold-standard” test is often not possible in the application of wildlife pathogen research and false-positive errors remain a constant concern. Poor specificity of diagnostic tests of bacteria in the *Mannheimia* genus has been previously documented [8]. We minimized false-positive detections due to poor specificity by generalizing our classification of bacteria in the *Mannheimia* genus to encompass the different species that could be mistaken for each other. The trade-off for this generalization is the potential violation of the third assumption of this modeling approach. Given these efforts to minimize false-positive errors and the findings of a recent study which found false-positive errors were relatively rare compared to false-negative errors [9], we feel that if false-positive detections did occur in this study, they were rare.

**References**

1. McClintock BT, Nichols JD, Bailey LL, MacKenzie DI, Kendall WL, Franklin AB. Seeking a second opinion: uncertainty in disease ecology. Ecol Lett. 2010;13: 659–674. doi:10.1111/j.1461-0248.2010.01472.x

2. Lachish S, Gopalaswamy AM, Knowles SCL, Sheldon BC. Site-occupancy modelling as a novel framework for assessing test sensitivity and estimating wildlife disease prevalence from imperfect diagnostic tests. Methods Ecol Evol. 2012;3: 339–348. doi:10.1111/j.2041-210X.2011.00156.x

3. Miller DAW, Talley BL, Lips KR, Campbell Grant EH. Estimating patterns and drivers of infection prevalence and intensity when detection is imperfect and sampling error occurs: Detection in epidemiological studies. Methods Ecol Evol. 2012;3: 850–859. doi:10.1111/j.2041-210X.2012.00216.x

4. Walsh DP, Wolfe LL, Vieira MEP, Miller MW. Detection probability and Pasteurellaceae surveillance in bighorn sheep. J Wildl Dis. 2012;48: 593–602.

5. MacKenzie DI, Nichols JD, Lachman GB, Droege S, Andrew Royle J, Langtimm CA. Estimating site occupancy rates when detection probabilities are less than one. Ecology. 2002;83: 2248–2255. Available: http://www.esajournals.org/doi/abs/10.1890/0012-9658(2002)083%5B2248:ESORWD%5D2.0.CO%3B2

6. Cassirer EF, Manlove KR, Plowright RK, Besser TE. Evidence for strain-specific immunity to pneumonia in bighorn sheep. J Wildl Manag. 2016; n/a-n/a. doi:10.1002/jwmg.21172

7. Royle JA, Link WA. Generalized site occupancy models allowing for false positive and false negative errors. Ecology. 2006;87: 835–841. doi:10.1890/0012-9658(2006)87[835:GSOMAF]2.0.CO;2

8. Miller MW, Hause BM, Killion HJ, Fox KA, Edwards WH, Wolfe LL. Phylogenetic and epidemiologic relationships among Pasteurellaceae from Colorado bighorn sheep herds. J Wildl Dis. 2013;49: 653–660. doi:10.7589/2012-11-274

9. Walsh DP, Cassirer EF, Bonds MD, Brown DR, Edwards WH, Weiser GC, et al. Concordance in diagnostic testing for respiratory pathogens of bighorn sheep. Wildl Soc Bull. 2016;40: 634–642. doi:10.1002/wsb.721
