# Supplementary material for: Assessing respiratory pathogen communities in bighorn sheep populations: Sampling realities, challenges, and improvements
Source: PLoS One. 2017 Jul 14;12(7):e0180689. doi: 10.1371/journal.pone.0180689 (PMC5510838; doi:10.1371/journal.pone.0180689)
Supplement: S2 Appendix — (DOCX) [file pone.0180689.s002.docx]

**S2 Appendix. Pathogen Diagnostic Protocol Descriptions**

***Pasteurellaceae***:

*Wyoming:*

Cultures from the tonsils were obtained using sterile polyester applicators (Puritan#25-806 1PD, Guilford, ME, USA) applied to the tonsilar crypts and the outer tonsil surface. Inoculated swabs were immediately used to inoculate one quarter of a Columbia Blood Agar plate (CBA) with 5 % sheep blood (Hardy Diagnostics #A16, Santa Maria, CA, USA). The applicator was then used to resample the tonsil, followed by placement into transport media; Port-A-Cul^TM^ tubes (Becton Dickinson, Franklin Lakes, NJ, USA). For ten animals where Port-A-Cul^TM^ tubes were not available, swabs were placed in 3mL Amies media without charcoal in a 15 x 103mm culture tube (Triforest Enterprises, Irvine, CA, USA). The inoculated swab was then placed into transport media as described above. All samples were transported to Wyoming Game and Fish Wildlife Health Laboratory and processed within four hours of collection. Bacterial plates were struck to three quadrants using a 1µm loop and incubated at 37^o^C in 5% CO_2_. Tonsil swabs were removed aseptically from the transport media with forceps and used to inoculate one half of a CBA plate. Tissues were aseptically removed from 18oz Whirl-Pak® bags (Nasco, Fort Atkinson, WI, USA), cut to expose an interior area of tissue, then smeared over half of a CBA plate. The plate was struck to two quadrants for isolation and incubated at 37⁰C in 5% CO_2_. Culture plates were read and documented once at ~18-24 hours, and again at ~36-48 hours. Targeted colonies were recultured for isolation and identified using standard biochemical tests [1]. After 48 hours, all bacterial growth on plate was collected with a polyester swab and placed in 15mL Falcon tubes (Corning, Corning, NY USA) filled with sterile phosphate buffered saline (BBL FTA Hemagglutination, Buffer Becton Dickinson, Franklin Lakes, NJ, USA), and vortexed to suspended bacteria. A 250µl aliquot was removed and placed into a PCR tube (PCR clean 1.5 mL safe-lock tubes Eppendorf, Hauppauge, NY USA) for DNA extraction (E.Z.N.A. Tissue DNA kit, Omega Bio-Tek, Norcross, Georgia, USA) per manufacturer’s instructions. Each sample was screened with PCR for the leukotoxin (*lktA*) gene with primers that amplified *lktA* in both *Mannheimia* species and *B. trehalosi* [2]. Positive samples were then analyzed using only the *Mannheimia lktA* gene PCR [3]. Samples positive on the initial PCR and negative on the second were categorized as *lktA* positive *B. trehalosi*. *Mannheimia* *spp* *lktA* assay will amplify *lktA* in *M. haemolytica, M. glucosida*, and *M.ruminalis*. *Mannheimia haemolytica /glucosdia lktA* specific PCR [4] was then performed on those samples positive for *Mannheimia* *lktA*.

*TSB:*

A single tonsil swab was collected from animals as described in the Wyoming protocol and placed immediately into a vial of tryptic soy broth (TSB). Samples were frozen as soon as possible and shipped overnight on dry ice to Washington Animal Disease Diagnostic Laboratory (WADDL) for *Pasteurellaceae* culture following the lab’s standard operating procedures. Swabs remained frozen at WADDL until they were plated by diagnosticians.

*Port-A-Cul:*

A single tonsil swab was collected as described, placed in a Port-A-Cul ™ tube and kept chilled until received by the diagnostic lab. Samples were shipped overnight on ice packs to WADDL for *Pasteurellaceae* culture following the lab’s standard operating procedures. Samples were shipped to WADDL as soon as possible, arriving within 72 hours of collection. Samples were plated by WADDL immediately upon receipt.

*Plated Culture:*

A single tonsil swab was collected as described and immediately used to inoculate a Columbia Blood Agar (CBA) culture plate with 5% sheep blood (Hardy Diagnostics, Santa Maria, California, USA) and a treated as described in the Wyoming PCR protocol. Following the Wyoming PCR protocol, the plate was struck to three quadrants for bacterial colony isolation the day of sample collection. After ~24 hours, a strip of the primary streak zone was swabbed with a sterile polyester tipped swab, as were any phenotypically distinct colonies present on the plate. This swab was placed immediately into a vial of TSB. Samples were frozen immediately and shipped overnight on dry ice to WADDL for *Pasteurellaceae* culture. Swabs remained frozen at WADDL until they were plated by diagnosticians.

*Plated PCR*

Following completion of the MSU protocol, CBA plates were incubated an additional ~24 hours before bacterial growth was cleared from the CBA plate as described in the Wyoming PCR protocol. Samples were stored at approximately - 20° C until being assessed by the Wyoming Game & Fish Department Wildlife Health Laboratory using the PCR procedures described in the Wyoming PCR protocol.

***Mycoplasma ovipneumoniae***

*Wyoming*

Samples from the nasal passages were obtained by inserting sterile polyester applicators (Puritan#25-806 1PD, Guilford, ME, USA) 8-12 cm into the nasal cavity and slowly rotating the shaft. The inoculated swab was then placed into transport media: Port-A-Cul^TM^ tubes (Becton Dickinson, Franklin Lakes, NJ, USA).For ten animals where Port-A-Cul^TM^ tubes were not available, swabs were placed in 3mL Amies media without charcoal in a 15 x 103mm culture tube (Triforest Enterprises, Irvine, CA, USA). Swabs were removed from the transport media as previously described and placed into 2mL tryptone soy broth (TSB-1) [5] with slight modifications [6] in sterile 5mL round-bottom tubes (BD Falcon, Franklin Lakes, NJ, USA) and incubated at 37°C with 5% CO_2_ for 48 hours. DNA was extracted from 1 mL of the TSB-1 as described for the Wyoming PCR *Pasteurellaceae* protocol. DNA was analyzed using primers and PCR protocol published by McAuliffe [7], and optimized in the Wyoming Game and Fish Department Wildlife Health lab by modifying the initial denaturation for five minutes at 94°C, 32 denaturation cycles for 30 seconds each at 94°C, annealing at 57.5°C for 30 seconds, and extension at 72°C for 30 seconds. The final extension was at 72°C for 5 minutes. Samples were kept at 4°C until analyzed.

*qPCR*

A nasal swab was collected as described in the Wyoming PCR protocol and placed in a sterile cryovial without transport media and stored frozen. The samples were shipped to WADDL on dry ice and were tested for presence of *Mycoplasma ovipneumoniae* using quantitative PCR (qPCR) to aid in protocol development. This does not represent a fee-for-service (FFS) protocol.

*TSB*

A nasal swab was collected as described in the Wyoming PCR protocol and placed immediately into a vial of tryptic soy broth (TSB). Samples were frozen as soon as possible and shipped overnight on dry ice to Washington Animal Disease Diagnostic Laboratory (WADDL) for *Mycoplasma ovipneumoniae* PCR testing [7,8].

**References**

1. Quinn PJ, Markey BK, Leonard FC, FitzPatrick ES, Fanning S, Hartigan P. Veterinary Microbiology and Microbial Disease. John Wiley & Sons; 2011.

2. Dassanayake RP, Shanthalingam S, Subramaniam R, Herndon CN, Bavananthasivam J, Haldorson GJ, et al. Role of Bibersteinia trehalosi, respiratory syncytial virus, and parainfluenza-3 virus in bighorn sheep pneumonia. Vet Microbiol. 2013;162: 166–172. doi:10.1016/j.vetmic.2012.08.029

3. Shanthalingam S, Goldy A, Bavananthasivam J, Subramaniam R, Batra SA, Kugadas A, et al. PCR assay detects Mannheimia haemolytica in culture-negative pneumonic lung tissues of bighorn sheep (Ovis canadensis) from outbreaks in the western USA, 2009-2010. J Wildl Dis. 2014;50: 1–10. doi:10.7589/2012-09-225

4. Angen O, Thomsen J, Larsen LE, Larsen J, Kokotovic B, Heegaard PMH, et al. Respiratory disease in calves: Microbiological investigations on trans-tracheally aspirated bronchoalveolar fluid and acute phase protein response. Vet Microbiol. 2009;137: 165–171. doi:10.1016/j.vetmic.2008.12.024

5. Patel H, Mackintosh D, Ayling RD, Nicholas RAJ, Fielder MD. A novel medium devoid of ruminant peptone for high yield growth of Mycoplasma ovipneumoniae. Vet Microbiol. 2008;127: 309–314. doi:10.1016/j.vetmic.2007.08.030

6. Jennings-Gaines J. Standard operating procedure: Procedures for isolating Mycoplasma ovipneumoniae. Wyoming Game and Fish Department, Wildlife Health Department; 2012. Available: https://wgfd.wyo.gov/WGFD/media/content/PDF/Vet%20Services/Procedures-for-Isolating-Mycoplasma-ovipneumoniae.pdf

7. McAuliffe L, Ellis RJ, Ayling RD, Nicholas RAJ. Differentiation of Mycoplasma species by 16S ribosomal DNA PCR and denaturing gradient gel electrophoresis fingerprinting. J Clin Microbiol. 2003;41: 4844–4847.

8. Lawrence PK, Shanthalingam S, Dassanayake RP, Subramaniam R, Herndon CN, Knowles DP, et al. Transmission of Mannheimia haemolytica from domestic sheep (Ovies aries) to bighorn sheep (Ovis canadensis): Unequivocol demonstration with green fluorescent protein-tagged organisms. J Wildl Dis. 2010;46: 706–717.
