# Supplementary material for: Assessing respiratory pathogen communities in bighorn sheep populations: Sampling realities, challenges, and improvements
Source: PLoS One. 2017 Jul 14;12(7):e0180689. doi: 10.1371/journal.pone.0180689 (PMC5510838; doi:10.1371/journal.pone.0180689)
Supplement: S3 Appendix — (DOCX) [file pone.0180689.s003.docx]

**S3 Appendix. Assessing inter-state differences in detection probability estimates**

Two subsets of data corresponding to samples obtained from bighorn sheep sampled for respiratory pathogens in Montana and bighorn sheep sampled in Wyoming were analyzed to assess whether detection probability estimates for each *Pasteurellaceae* pathogen differed between states. The *Pasteurellaceae* TSB protocol was conducted on 456 of 476 individual bighorn sheep in the dataset, whereas other protocols were all conducted on less than 50% of the sampled individuals (Table S2.1). Similarly, the *Mycoplasma ovipneumoniae* TSB protocol was conducted on 431 of the 466 individuals sampled for *Mycoplasma ovipneumoniae* (Table S2.2)*.* No other *Mycoplasma ovipneumoniae* protocol was conducted on more than 133 individuals. Therefore, if detection probability of the TSB protocols varied across states, there was potential for inaccurate estimates of detection probability for the other protocols, which were typically conducted in tandem with the TSB protocol. Accordingly, the focus of this procedure was to estimate detection probability of each diagnostic protocol for *Pasteurellaceae* and *Mycoplasma ovipneumoniae* using only subsets of data where both that protocol and the TSB protocol were conducted on all animals and compare the resulting estimates to that obtained from analysis of the complete dataset. The Port-A-Cul *Pasteurellaceae* protocol was not assessed here because it was not conducted a sufficient number of times.

The first *Pasteurellaceae* subset (Montana) included only data from bighorn sheep sampled in Montana, where samples from each animal were collected and tested using the TSB, Plated Culture, and Plated PCR protocols (n=152 individual animals). The Plated-PCR protocol did not assess presence of *Pasteurella multocida*, but this pathogen’s detection probability using the TSB and Plated Culture protocols was still assessed in this dataset. Detection probability for *Bibersteinia trehalosi* was not assessed in this dataset due to insufficient detections (n=2). The second *Pasteurellaceae* subset (Wyoming) included data from 122 bighorn sheep sampled in Wyoming, where samples were collected and tested using the *Pasteurellaceae* Wyoming and TSB protocols. For *Mycoplasma ovipneumoniae,* the Montana subset included data from 106 animals where two samples were assessed from each animal using the TSB protocol and the Wyoming subset included data from 95 animals where samples were assessed using the Wyoming and TSB protocols.

The pathogen-specific subsets were then filtered to exclude population-years (i.e. a given population in a given year) where that pathogen was not detected in order to minimize model convergence issues associated with estimating pathogen prevalence parameters at a boundary. The same occupancy model structure was applied to all the datasets to assess whether estimates of detection probability varied across the subsets of data. The occupancy model used for this assessment allowed each pathogen’s estimated prevalence to vary by population-year and allowed each pathogen’s estimated detection probability (ρ) to vary by diagnostic protocol. When detection probability estimates approached zero or one, boundary issues prevented the model from accurately estimating standard errors. This situation occurred when a protocol never detected a pathogen or when a protocol never failed to detect a pathogen in an animal known to be infected. In these cases, no standard errors are shown in Fig. S3.1.

For nearly every combination of pathogen and protocol, the parameter estimates for detection probability were similar between the subsets and the complete set (μ _difference_ = 0.04) and confidence intervals overlapped substantially (Fig. S3.1); however, detection probability estimates for the TSB protocol clearly differed between the datasets for *Mannheimia spp.* (Fig. S3.1). The detection probability estimate for this pathogen-protocol combination obtained from the complete dataset was 0.12 (95% CI: 0.08-0.16); the estimate from the Wyoming subset was 0.31 (95% CI: 0.21-0.43); and the estimate from the Montana subset was 0.01 (95% CI: 0.00-0.06).

After confirming that the detection probability estimates for the other diagnostic protocols used to detect *Mannheimia spp.* were not strongly affected by the disparate estimates of detection probability for the TSB protocol across the two states (Fig. S3.1), we kept the *Mannheimia spp.* detection probability estimates from the full dataset (as opposed to creating state-specific estimates) based on the fact that there is no clear methodological explanation for the heterogeneity of the TSB protocol, the estimates of detection probability for the other protocols were similar between the subsets and the complete dataset, and that our objectives require a single estimate of detection probability for each protocol.

The difference in detection probability for *Mannheimia spp.* between the two states using the TSB protocol may be explained by animals sampled in the two states hosting different species within the *Mannheimia* genus. This assertion is supported by the observation that in winter 2015/2016 (the only year when *Mannheimia ruminalis* could be distinguished) most *Mannheimia spp.* isolates (as defined in our analysis) from Montana were identified by WADDL as *Mannheimia ruminalis* while most isolates from Wyoming were identified by WADDL as unidentified *Mannheimia species.* The detection power estimates we provide for *Mannheimia spp.* using the TSB protocol represent a compromise between two disparate estimates we obtained for samples collected in different state; thus additional investigation using diagnostic tests with improved specificity is needed to provide better sampling recommendations for this class of pathogens.

Table S2.1. Number of animals sampled in each study population and year for *Pasteurellaceae* pathogens and the mean number of times each protocol was conducted per animal in parentheses. The total number of animals sampled and mean number of total diagnostic protocols conducted per animal are also shown in the total column.

| Population-Year | TSB | Port-A-Cul | Plated PCR | Plated Culture | Wyoming | **TOTAL** |
| --- | --- | --- | --- | --- | --- | --- |
| Castle Reef 14.15 | 23 (1) |  | 21 (1) | 16 (1) |  | 23 (2.61) |
| Castle Reef 15.16 | 7 (2) |  | 7 (1) | 7 (2) |  | 7 (5) |
| Devil's Canyon 15.16 | 22 (1) |  |  |  | 25 (1) | 25 (1.88) |
| Dubois Badlands 15.16 | 4 (1) |  |  |  | 5 (1) | 5 (1.8) |
| Mt Everts 13.14 | 5 (1) |  |  |  |  | 5 (1) |
| Fergus 14.15 | 60 (1) |  | 29 (1) | 15 (1) |  | 60 (1.73) |
| Highlands 15.16 | 16 (2) |  |  |  |  | 16 (2) |
| Hilgard 13.14 | 29 (1) |  |  |  |  | 29 (1) |
| Hilgard 14.15 | 49 (1.98) |  | 38 (1) | 18 (1) |  | 49 (3.12) |
| Hilgard 15.16 | 34 (1.97) | 34 (1) | 34 (1) | 31 (1.97) |  | 34 (5.77) |
| Jackson 15.16 | 16 (1) |  |  |  | 16 (1) | 16 (2) |
| Lost Creek 14.15 | 13 (1) |  | 13 (1) | 13 (1) |  | 13 (3) |
| Lost Creek 15.16 | 6 (2) |  | 5 (1) | 6 (2) |  | 6 (4.83) |
| Middle Missouri Breaks 15.16 | 19 (2) |  |  |  |  | 19 (2) |
| Perma-Paradise 14.15 | 30 (1) |  | 28 (1) | 15 (1) |  | 30 (2.43) |
| Petty Creek 15.16 | 16 (2) | 17 (1) | 17 (1) | 17 (2) |  | 17 (5.88) |
| Stillwater 14.15 | 16 (1) |  | 16 (1) | 16 (1) |  | 16 (3) |
| Sybille 15.16 | 11 (2) | 11 (1) |  | 11 (1) | 11 (1) | 11 (5) |
| Temple Peak 15.16 | 9 (1) |  |  |  | 14 (1) | 14 (1.64) |
| Trout Peak 15.16 | 7 (1) |  |  |  | 8 (1) | 8 (1.88) |
| Wapiti Ridge 12.13 | 16 (1) |  |  |  | 16 (1) | 16 (2) |
| Wapiti Ridge 14.15 | 7 (1) |  |  | 7 (1) | 7 (1) | 7 (3) |
| Wapiti Ridge 15.16 | 10 (1) |  |  |  | 15 (1) | 15 (1.67) |
| Whiskey Mountain 15.16 | 12 (1) |  |  |  | 8 (1) | 13 (1.54) |
| Not Analyzed^1^ | 19 (1.47) |  | 4 (1) | 1 (2) | 11 (1) | 22 (2.18) |
| **TOTAL** | 456 (1.36) | 62 (1) | 212 (1) | 176 (1.34) | 136 (1) | **476 (2.66)** |

^1^ Population –years where less than five individual bighorn sheep were sampled (n=10) were not considered in analysis

Table S2.2. Number of animals sampled in each study population and year for *Mycoplasma ovipneumoniae* and the mean number of times each protocol was conducted per animal in parentheses. The total number of animals sampled and mean number of total diagnostic protocols conducted per animal are also shown in the total column.

| Population-Year^1^ | TSB | qPCR | Wyoming | **TOTAL** |
| --- | --- | --- | --- | --- |
| Castle Reef 14.15 | 23 (1) | 15 (1) |  | 23 (1.65) |
| Castle Reef 15.16 | 7 (2) |  |  | 7 (2) |
| Devil's Canyon 15.16 |  |  | 25 (1) | 25 (1) |
| Dubois Badlands 15.16 | 5 (1) |  | 5 (1) | 5 (2) |
| Mt Everts 13.14 | 5 (1) |  |  | 5 (1) |
| Fergus 14.15 | 59 (1) | 29 (1) |  | 59 (1.49) |
| Highlands 15.16 | 16 (2) |  |  | 16 (2) |
| Hilgard 13.14 | 29 (1) |  |  | 29 (1) |
| Hilgard 14.15 | 50 (1) |  |  | 50 (1) |
| Hilgard 15.16 | 35 (2) |  |  | 35 (2) |
| Jackson 15.16 | 16 (1) |  | 16 (1) | 16 (2) |
| Lost Creek 14.15 | 13 (1) | 7 (1) |  | 13 (1.54) |
| Lost Creek 15.16 | 6 (2) |  |  | 6 (2) |
| Middle Missouri Breaks 15.16 | 19 (2) |  |  | 19 (2) |
| Paradise 14.15 | 30 (1) | 30 (1) |  | 30 (2) |
| Petty Creek 15.16 | 16 (2) |  |  | 16 (2) |
| Stillwater 14.15 | 16 (1) | 7 (1) |  | 16 (1.44) |
| Sybille 15.16 | 11 (2) |  | 11 (1) | 11 (3) |
| Temple Peak 15.16 | 12 (1) |  | 14 (1) | 14 (1.86) |
| Trout Peak 15.16 | 8 (1) |  | 8 (1) | 8 (2) |
| Wapiti Ridge 12.13 | 16 (1) |  | 16 (1) | 16 (2) |
| Wapiti Ridge 14.15 |  |  | 7 (1) | 7 (2) |
| Wapiti Ridge 15.16 | 14 (1) |  | 15 (1) | 15 (1.93) |
| Whiskey Mountain 15.16 | 8 (1) |  | 8 (1) | 8 (2) |
| Not Analyzed^1^ | 17 (1.41) |  | 8 (1) | 20 (1.60) |
| **TOTAL** | 431 (1.27) | 88 (1) | 133 (1) | **469 (1.64)** |

^1^ Population –Years where less than five individual bighorn sheep were sampled (n=10) were not considered in analysis.


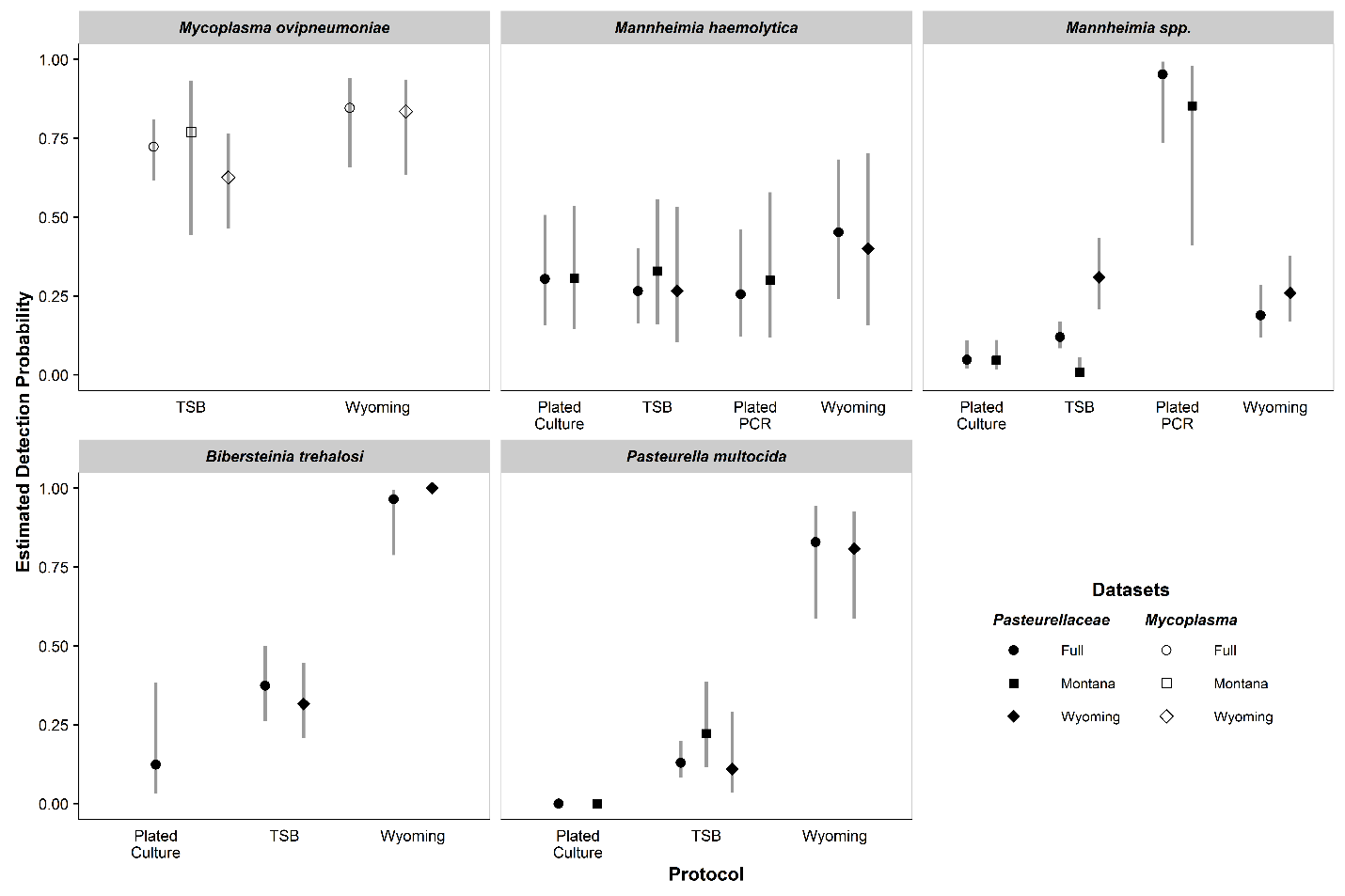


Fig. S3.1. Estimated detection probabilities and 95% confidence intervals for bighorn sheep respiratory pathogens obtained from independent analyses of the complete dataset and two independent subsets defined by the state where data were collected (Montana or Wyoming). The subsets only included data from bighorn sheep where multiple protocols were conducted. The different diagnostic protocols are shown on the x-axis of each facet and the detection probability estimates obtained for each protocol from analysis of the different datasets are shown adjacent to each other in different shapes. The occupancy model structure used to obtain these estimates allowed pathogen prevalence to vary by population and year and detection probability to vary by protocol.
