# Supplementary material for: Assessing respiratory pathogen communities in bighorn sheep populations: Sampling realities, challenges, and improvements
Source: PLoS One. 2017 Jul 14;12(7):e0180689. doi: 10.1371/journal.pone.0180689 (PMC5510838; doi:10.1371/journal.pone.0180689)
Supplement: S4 Appendix — (DOCX) [file pone.0180689.s004.docx]

**S4 Appendix. Derivation of Detection Power Solution**

Estimates of detection probability (conditional on infection) for a single sample were first scaled to the level of the individual with multiple samples, and then scaled to the population level to assess the probability of at least one detection in an infected population.

We used a hierarchical model to calculate the probability of at least one detection in a single, infected individual with multiple samples collected. Momentarily suppressing subscripts for different pathogens and protocols, let the probability of detection for a particular pathogen on a single sample be ρ, which we modeled as a random variable with a $\mathrm{Beta}\left( \alpha, \beta\right)$ distribution. We modeled the number of positive samples (Y) obtained from an infected individual as a binomial distribution defined by the number of samples collected (s) and the probability of success. We can express the model as:

$$Y | p \sim\mathrm{Binomial}\left( s, p \right),$$

$$p \sim\mathrm{Beta}\left( \alpha, \beta\right)$$

Following the approach of Walsh et al. 2012 [1], we derived the marginal distribution of Y_ij_ as a betabinomial distribution and calculated the conditional probability of *not* obtaining at least one positive detection of the *i^th^* pathogen using the *j^th^* protocol (Y*_ij_* =0) in an *infected* individual as:

$$\Pr{(Y}_{ij}=0) =\frac{Beta\left( a_{ij}, s_{ij}+\beta_{ij} \right)}{Beta\left( a_{ij}, \beta_{ij} \right)},$$

where α_ij_ and β_ij_ represent the parameters of the beta distribution of the conditional detection probability for the *i^th^* pathogen using the *j^th^* protocol and s_ij_ represents the the number of samples collected from the individual and tested for the *i^th^* pathogen using the *j^th^* protocol.

We incorporated our estimates of detection probability for each pathogen-protocol combination into this expression by first creating a logit-normal distribution using the detection parameter estimate and associated standard error as the mean and standard deviation of the distribution. The distribution was then transformed to a beta-distribution that could be used to solve the equation by using maximum-likelihood estimation via the “fitdistrplus” package [2] in Program R [3]. Any detection probabilities that were estimated on a boundary were not incorporated into the subsequent detection probability analysis. Comparison of the quantiles of the model-estimated detection probability distributions and the corresponding derived beta distributions confirmed the corresponding distributions matched each other well (Table S4.1).

We departed from the approach of Walsh *et al.* [1] when we scaled the per-animal probability of detecting a pathogen to the population-level, specifically in our consideration of pathogen prevalence in a population. Where Walsh et al. modeled the number of infected animals that are sampled as a binomial distribution and marginalized over that distribution, we modeled the number of infected animals as a hypergeometric distribution and did not marginalize; Thus, our estimated probability of detecting a pathogen at the population-level is conditional on a specified pathogen prevalence and the corresponding probability estimated by Walsh et al. is not conditional on prevalence.

Conditional on prevalence, or the probability that any one individual is infected, the power to detect pathogen *i* in sampled population *w* depends on the probability of detecting pathogen *i* in each infected animal, as well as the number of infected animals (*I_iw_*_­_) that are sampled, the latter of which follows a hypergeometric distribution[4]. The use of the hypergeometric is particularly important for smaller populations. Let $N_{ij}$ be the number of infected individuals who return at least one positive for the *i*^th^ pathogen using the *j*^th^ protocol, and *I*_iw_ the number of sampled individuals with the *i*^th^ pathogen in the *w*^th^ population. We calculated the joint probability of *not* detecting the *i^th^* pathogen in population *w* while sampling $I$ infected animals using the *j^th^* protocol as:

$$\Pr\left( {N_{ij}=0, I}_{iw} | \psi_{iw},a_{ij},\beta_{ij} \right) =\Pr\left( N_{ij}=0 | I_{iw} \right)\times\Pr\left( I_{iw} \right)$$

$$\Pr\left( {N_{ij}=0, I}_{iw} | \psi_{iw}{, a}_{ij},\beta_{ij} \right) =\left[ \frac{Beta\left( a_{ij}, s_{ij}+\beta_{ij} \right)}{Beta\left( a_{ij}, \beta_{ij} \right)} \right]^{I_{iw}}\times\frac{\left( \begin{matrix} N_{w}\psi_{iw} \\ I_{iw} \end{matrix} \right)\left( \begin{matrix} N_{w}-N_{w}\psi_{iw} \\ n_{w}-I_{iw} \end{matrix} \right)}{\left( \begin{matrix} N_{w} \\ n_{w} \end{matrix} \right)}$$

where $N_{w}$ equals the number of animals in population *w,* $\psi_{iw}$ is the prevalence of pathogen *i* in population *w*, $N_{w}\psi_{iw}$ is the number of animals infected by pathogen *i* in population *w,* and $n_{w}$ is the number of animals sampled in population *w.* When $N_{w}\psi_{iw}$was not an integer, the value was rounded down to the next integer. This probability statement was marginalized over *I_iw­_* to provide the following solution for the probability of not detecting pathogen *i* in any animal using protocol *j*, after sampling *n* animals from population *w*.

$$\Pr\left( N_{ij}=0 \right)=\sum_{I_{iw}=0}^{\min{(n}_{w}, N_{w}\psi_{iw})} \left[ \frac{Beta\left( a_{ij}, s_{ij}+\beta_{ij} \right)}{Beta\left( a_{ij}, \beta_{ij} \right)} \right]^{I_{iw}}\times\frac{\left( \begin{matrix} N_{w}\psi_{iw} \\ I_{iw} \end{matrix} \right)\left( \begin{matrix} N_{w}-N_{w}\psi_{iw} \\ n_{w}-I_{iw} \end{matrix} \right)}{\left( \begin{matrix} N_{w} \\ n_{w} \end{matrix} \right)}$$

Accordingly, the complementary probability of detecting pathogen *i* in at least one sample, or $\Pr\left( N_{ij}>0 \right)$, after sampling *n* animals from population *w* using protocol *j* is:

$$\Pr\left( N_{ij}>0 \right)=1-\sum_{I_{iw}=0}^{\min{(n}_{w}, N_{w}\psi_{iw})} \left[ \frac{Beta\left( a_{ij}, s_{ij}+\beta_{ij} \right)}{Beta\left( a_{ij}, \beta_{ij} \right)} \right]^{I_{iw}}\times\frac{\left( \begin{matrix} N_{w}\psi_{iw} \\ I_{iw} \end{matrix} \right)\left( \begin{matrix} N_{w}-N_{w}\psi_{iw} \\ n_{w}-I_{iw} \end{matrix} \right)}{\left( \begin{matrix} N_{w} \\ n_{w} \end{matrix} \right)}$$

This result was then used in our power calculations of the probabilities of detection given the population size, prevalence rate, number of animals sampled, and samples collected per animal.

Table S4.1. Distribution parameters and quantiles obtained directly from model estimates of detection probability (Logit-Normal) for

each pathogen-protocol combination (not estimated on a boundary) and the corresponding derived beta distributions that were used to

assess detection power.

| **Pathogen** | **Protocol** | **Distribution Parameters** | | | **25% Quantile** | | **50% Quantile** | | **75% Quantile** | |
| --- | --- | --- | --- | --- | --- | --- | --- | --- | --- | --- |
|  |  | *Logit-Normal*  (μ,σ^2^) | *Beta*  (α, β) | | Logit-Normal | Beta | Logit-Normal | Beta | Logit-Normal | Beta |
| *Mannheima haemolytica* | |  |  | |  |  |  |  |  |  |
| Plated PCR | | -1.07, 0.46 | 6.63, 18.36 | | 0.201 | 0.202 | 0.256 | 0.259 | 0.319 | 0.321 |
| Plated Culture | | -0.83, 0.44 | 8, 17.65 | | 0.246 | 0.247 | 0.304 | 0.307 | 0.370 | 0.371 |
| Port-A-Cul | | -2.18, 1.07 | 1.22, 7.34 | | 0.052 | 0.055 | 0.101 | 0.115 | 0.189 | 0.203 |
| TSB | | -1.02, 0.32 | 14.01, 37.82 | | 0.226 | 0.227 | 0.266 | 0.267 | 0.310 | 0.310 |
| Wyoming PCR | | -0.19, 0.49 | 8.08, 9.71 | | 0.372 | 0.373 | 0.452 | 0.452 | 0.534 | 0.533 |
| *Mannheimia spp.* | |  |  | |  |  |  |  |  |  |
| Plated PCR | | 3.01, 1.02 | 16.11, 1.22 | | 0.911 | 0.901 | 0.953 | 0.946 | 0.976 | 0.974 |
| Plated Culture | | -2.99, 0.46 | 5.13, 93.05 | | 0.035 | 0.036 | 0.048 | 0.049 | 0.064 | 0.065 |
| TSB | | -2, 0.2 | 27.96, 202.63 | | 0.106 | 0.106 | 0.120 | 0.120 | 0.135 | 0.135 |
| Wyoming | | -1.46, 0.28 | 16.66, 69.99 | | 0.162 | 0.163 | 0.189 | 0.190 | 0.219 | 0.219 |
| *Bibersteinia trehalosi* | |  |  | |  |  |  |  |  |  |
| Plated Culture | | -2.15, 0.75 | 2.26, 15.79 | | 0.066 | 0.068 | 0.105 | 0.112 | 0.162 | 0.168 |
| TSB | | -0.59, 0.26 | 23.28, 41.64 | | 0.317 | 0.318 | 0.356 | 0.357 | 0.398 | 0.398 |
| Wyoming | | 3.29, 1.02 | 20.42, 1.2 | | 0.931 | 0.923 | 0.964 | 0.958 | 0.982 | 0.980 |
| *Pasteurella multocida* | |  |  | |  |  |  |  |  |  |
| Port-A-Cul | | -0.25, 1.29 | 1.48, 1.77 | | 0.246 | 0.258 | 0.437 | 0.444 | 0.650 | 0.642 |
| TSB | | -1.9, 0.26 | 16.84, 110.23 | | 0.111 | 0.111 | 0.130 | 0.131 | 0.151 | 0.152 |
| Wyoming | | 1.58, 0.63 | 14.48, 3.36 | | 0.761 | 0.756 | 0.829 | 0.823 | 0.882 | 0.879 |
| *Mycoplasma ovipneumoniae* | |  |  | |  |  |  |  |  |  |
| qPCR | | 0.59, 0.69 | 6.28, 3.69 | | 0.531 | 0.530 | 0.643 | 0.638 | 0.742 | 0.738 |
| TSB | | 0.98, 0.25 | | 60.18, 22.91 | 0.693 | 0.692 | 0.727 | 0.726 | 0.759 | 0.758 |
| Wyoming PCR | | 1.85, 0.53 | | 25, 4.36 | 0.815 | 0.812 | 0.864 | 0.859 | 0.901 | 0.899 |

**References**

1. Walsh DP, Wolfe LL, Vieira MEP, Miller MW. Detection probability and Pasteurellaceae surveillance in bighorn sheep. J Wildl Dis. 2012;48: 593–602.

2. Delignette-Muller ML, Dutang C, others. fitdistrplus: An R package for fitting distributions. J Stat Softw. 2015;64: 1–34. Available: http://cran.dataguru.cn/web/packages/fitdistrplus/vignettes/paper2JSS.pdf

3. R Core Team. R: a language and environment for statistical computing. R Foundation for Statisical Development; 2016.

4. Cameron AR, Baldock FC. A new probability formula for surveys to substantiate freedom from disease. Prev Vet Med. 1998;34: 1–17. doi:10.1016/S0167-5877(97)00081-0
