# Supplementary material for: Assessing respiratory pathogen communities in bighorn sheep populations: Sampling realities, challenges, and improvements
Source: PLoS One. 2017 Jul 14;12(7):e0180689. doi: 10.1371/journal.pone.0180689 (PMC5510838; doi:10.1371/journal.pone.0180689)

**S5 Appendix. Detection Power Curves for All Pathogen-Protocol Combinations**

These charts are intended to provide readers the ability to investigate suitable sampling methodologies and intensities based on their capabilities, restrictions, and preferences. Each page in this appendix illustrates the effects of various factors on the power to detect a pathogen in a sampled population, given the pathogen and the protocol used, which is stated at the top of the graphic, along with the estimated detection probability for that pathogen-protocol combination. Protocols that use a diagnostic test offered by a fee-for-service (FFS) laboratory are indicated with an asterisk following their name. Each page is split into quadrants to illustrate relationships under four different population sizes (N=25, 50, 100, & 200). Each quadrant is split into nine panels to show expected relationships through a sequence of pathogen prevalence ranging from 0.1 to 0.9. Each panel shows the power to detect the pathogen (y-axis), given the number of animals sampled (x-axis) and three lines are used to show this relationship when protocol are conducted one, two, and three times per animal. The horizontal dashed line in each panels corresponds with 80% power to detect the pathogen. The x-axes in the quadrant illustrating these relationships for a population size of 25 differs from the x-axes of the other quadrants. Power curves for several pathogen-protocol combination are not shown in the appendix because the protocol never successfully detected that particular pathogen. Pages are organized by pathogen family (*Pasteurellaceae* or *Mycoplasma*) and then by Protocols.

Table of Contents

[***Mycoplasma ovipneumoniae*** 2](#_Toc459388580)

[*TSB** 2](#_Toc459388581)

[*Wyoming* 4](#_Toc459388583)

[***Pasteurellaceae*** 5](#_Toc459388584)

[*TSB** 5](#_Toc459388585)

[*Port-A-Cul** 5](#_Toc459388586)

[*Plated Culture** 11](#_Toc459388587)

[*Plated PCR* 14](#_Toc459388588)

[*Wyoming* 16](#_Toc459388589)

# ***Mycoplasma ovipneumoniae***

## *TSB**


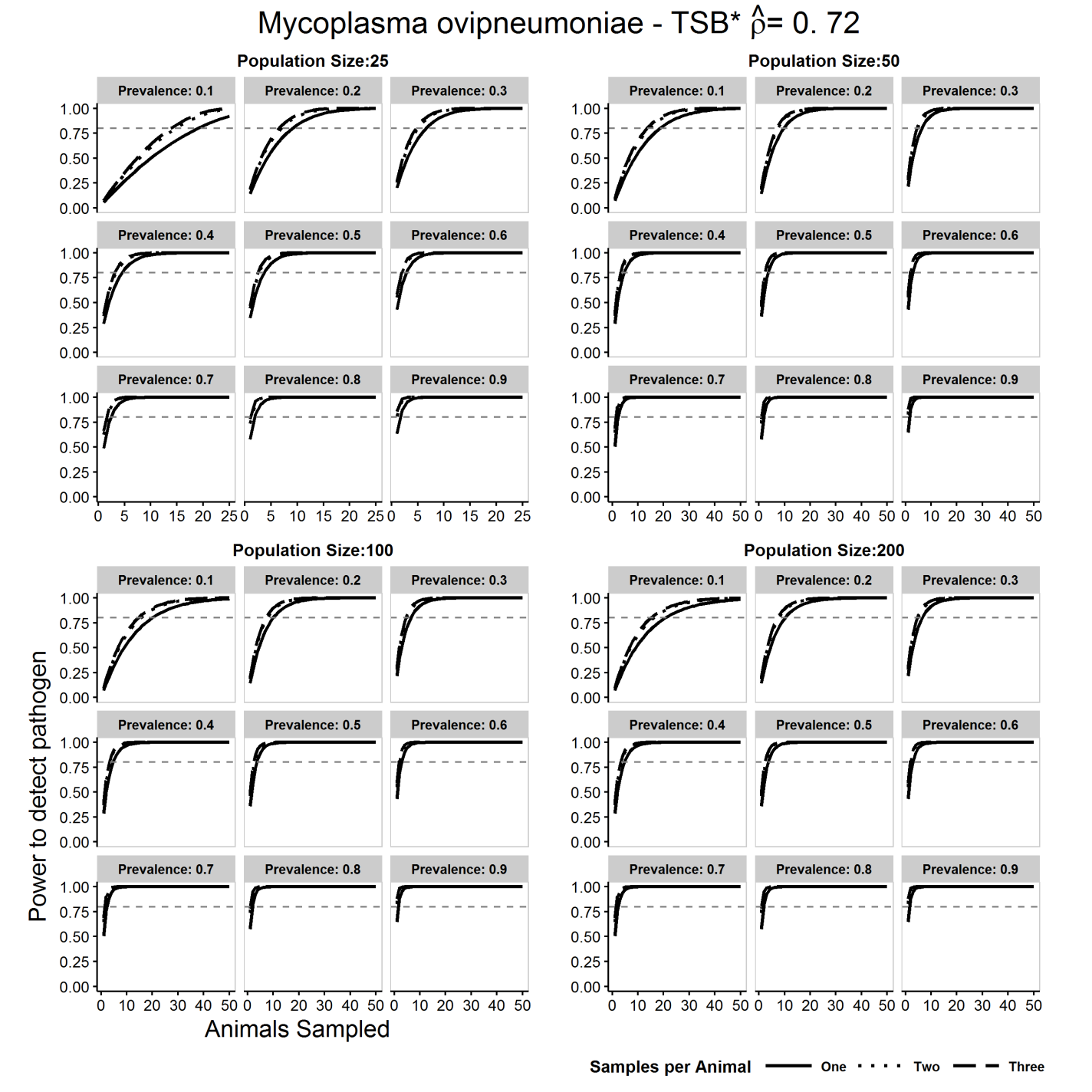


## *Wyoming*

*
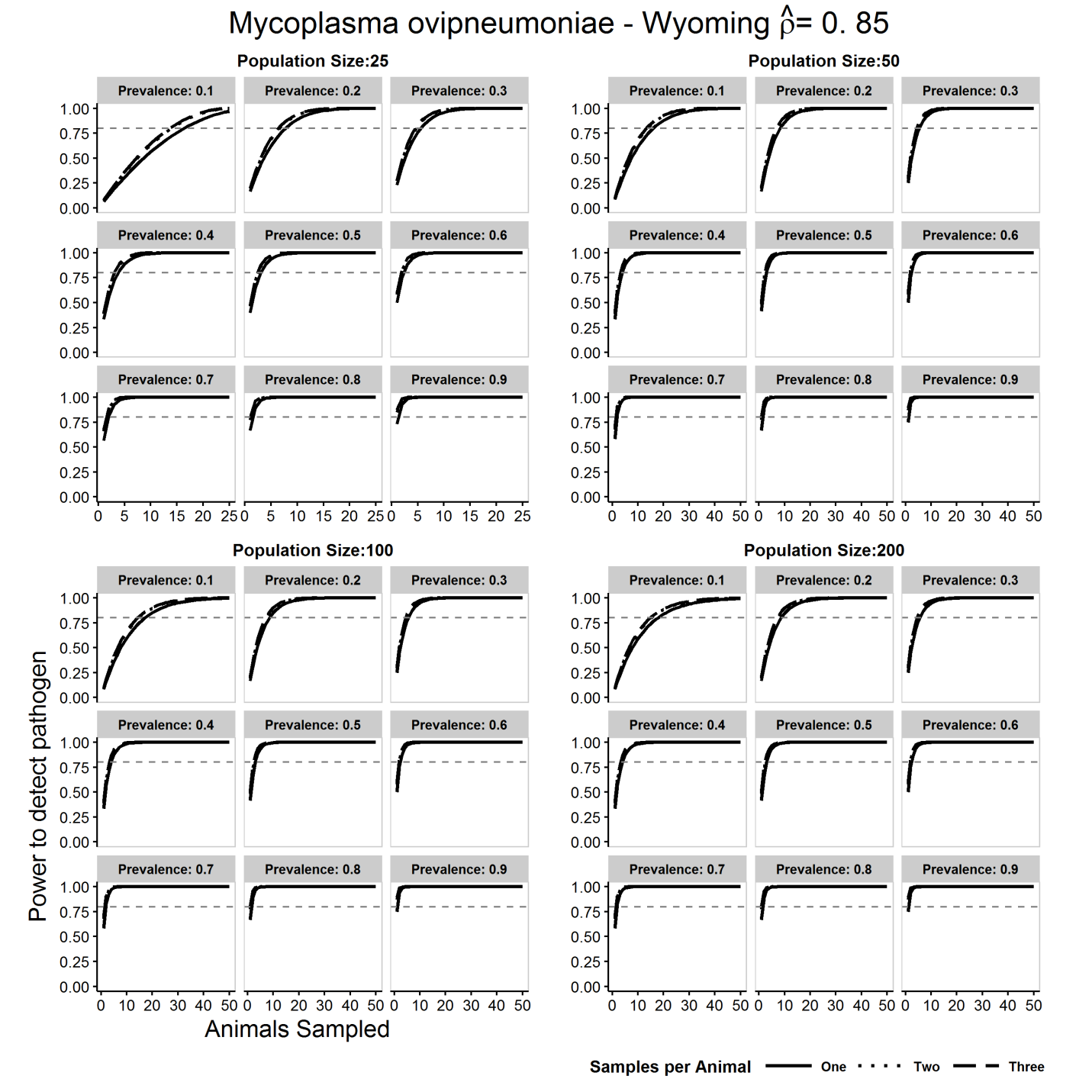
*

# ***Pasteurellaceae***

## *TSB**

## *
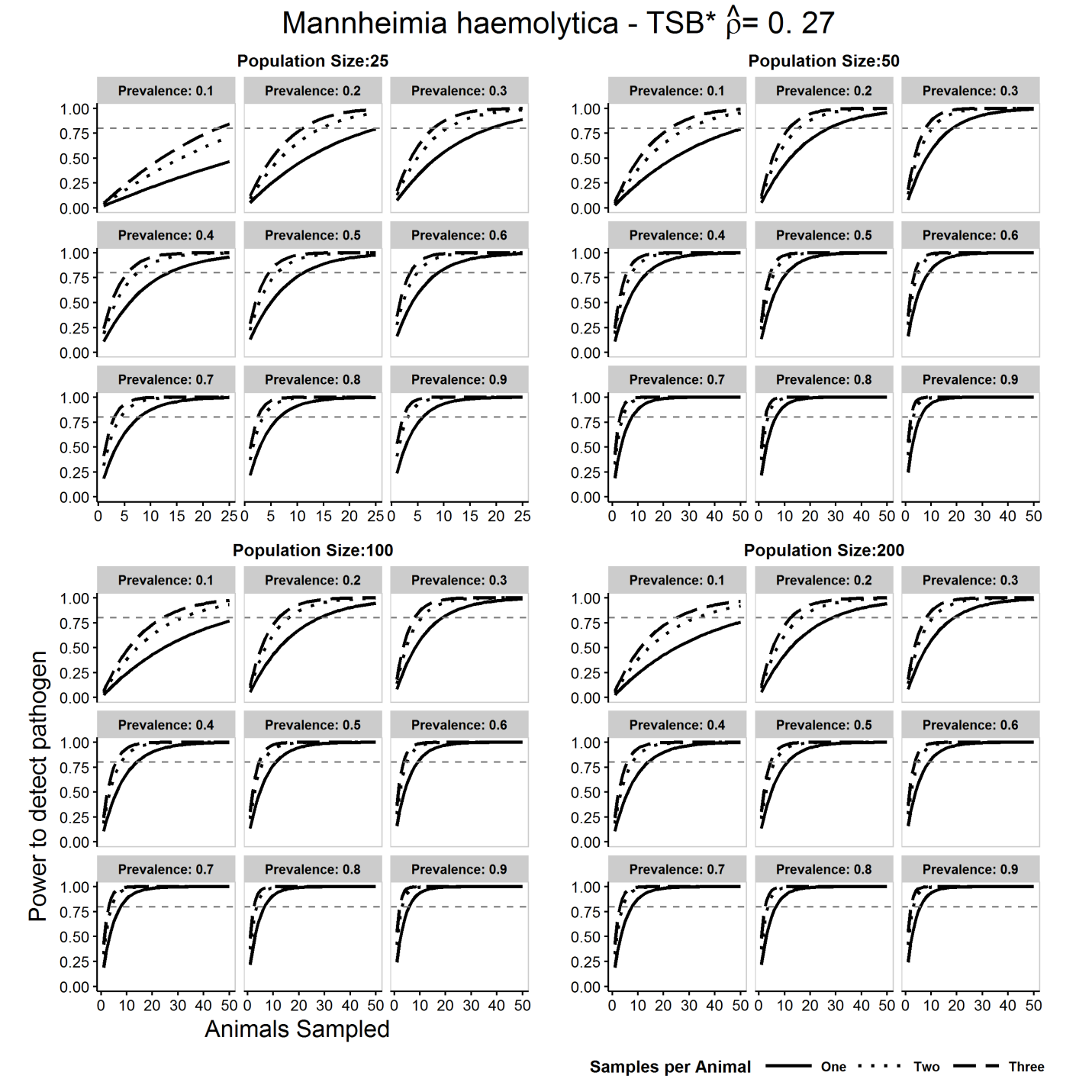
*


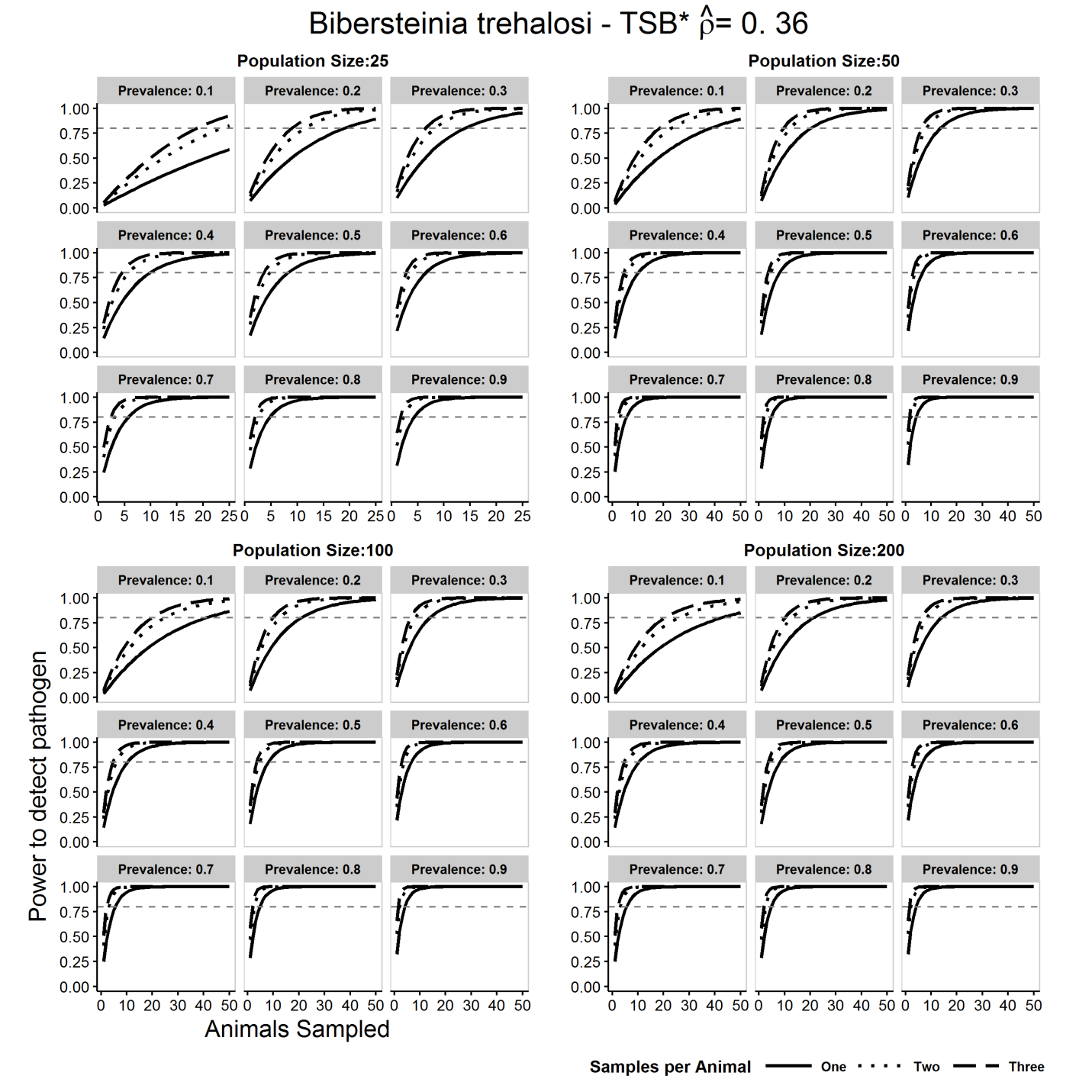


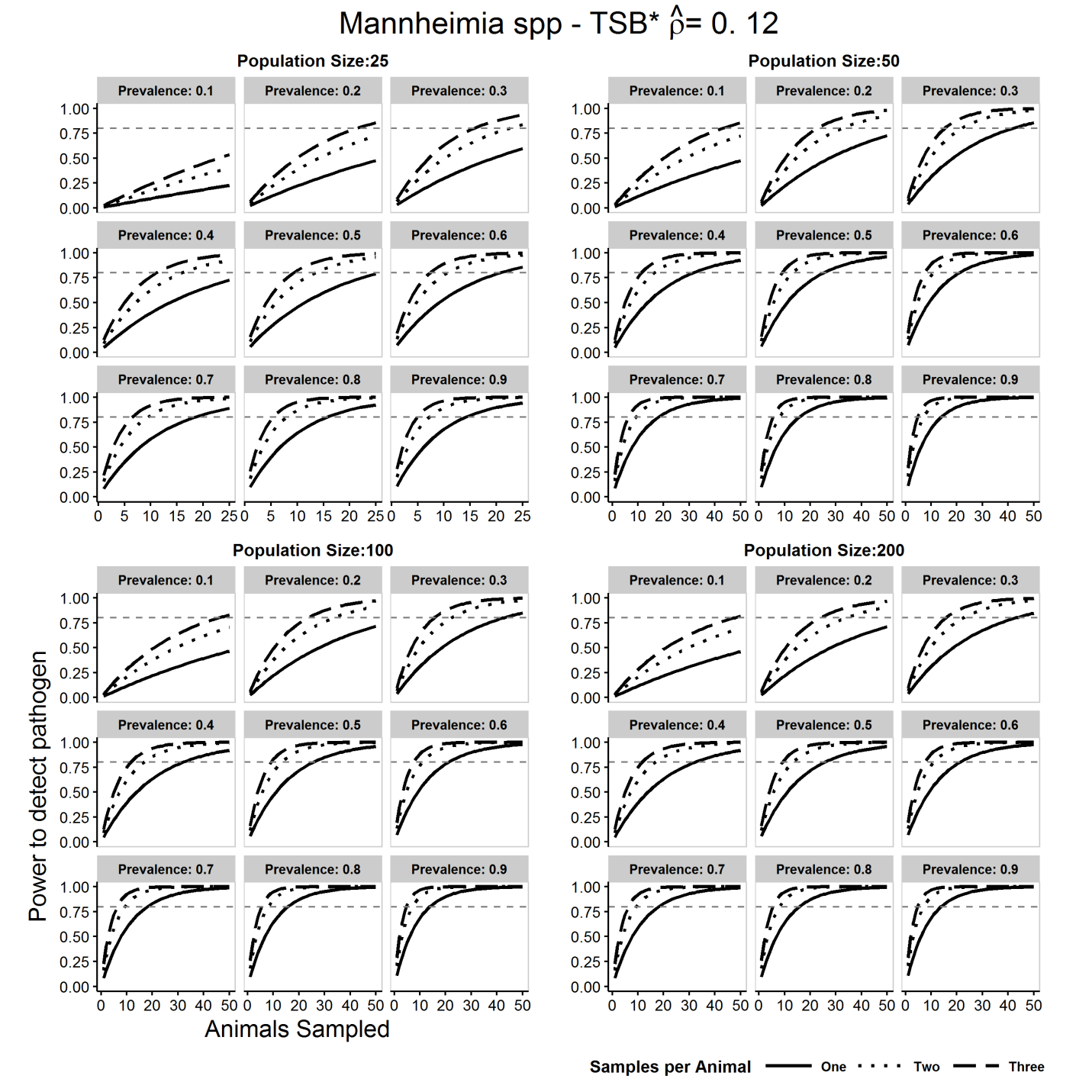


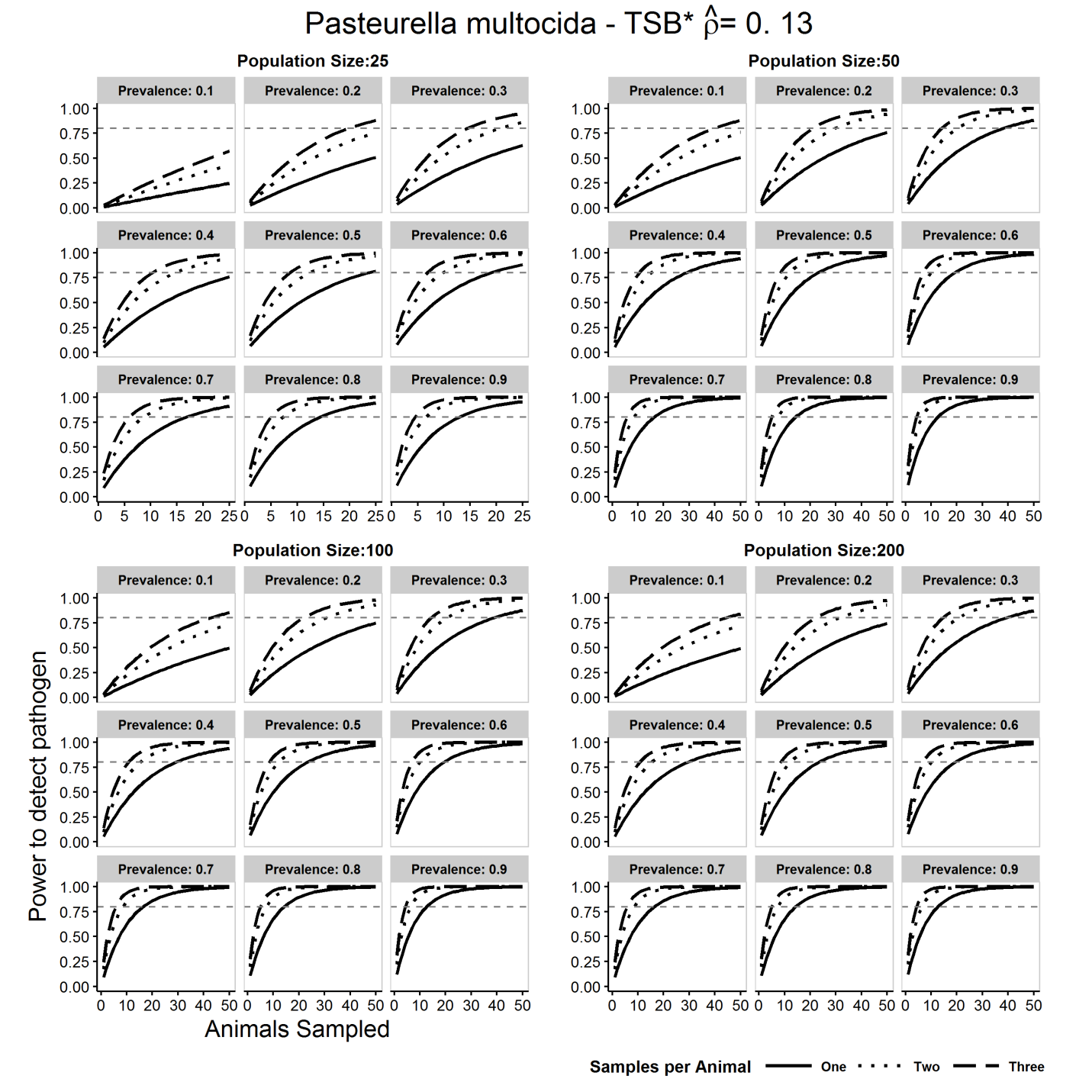


## *Port-A-Cul**


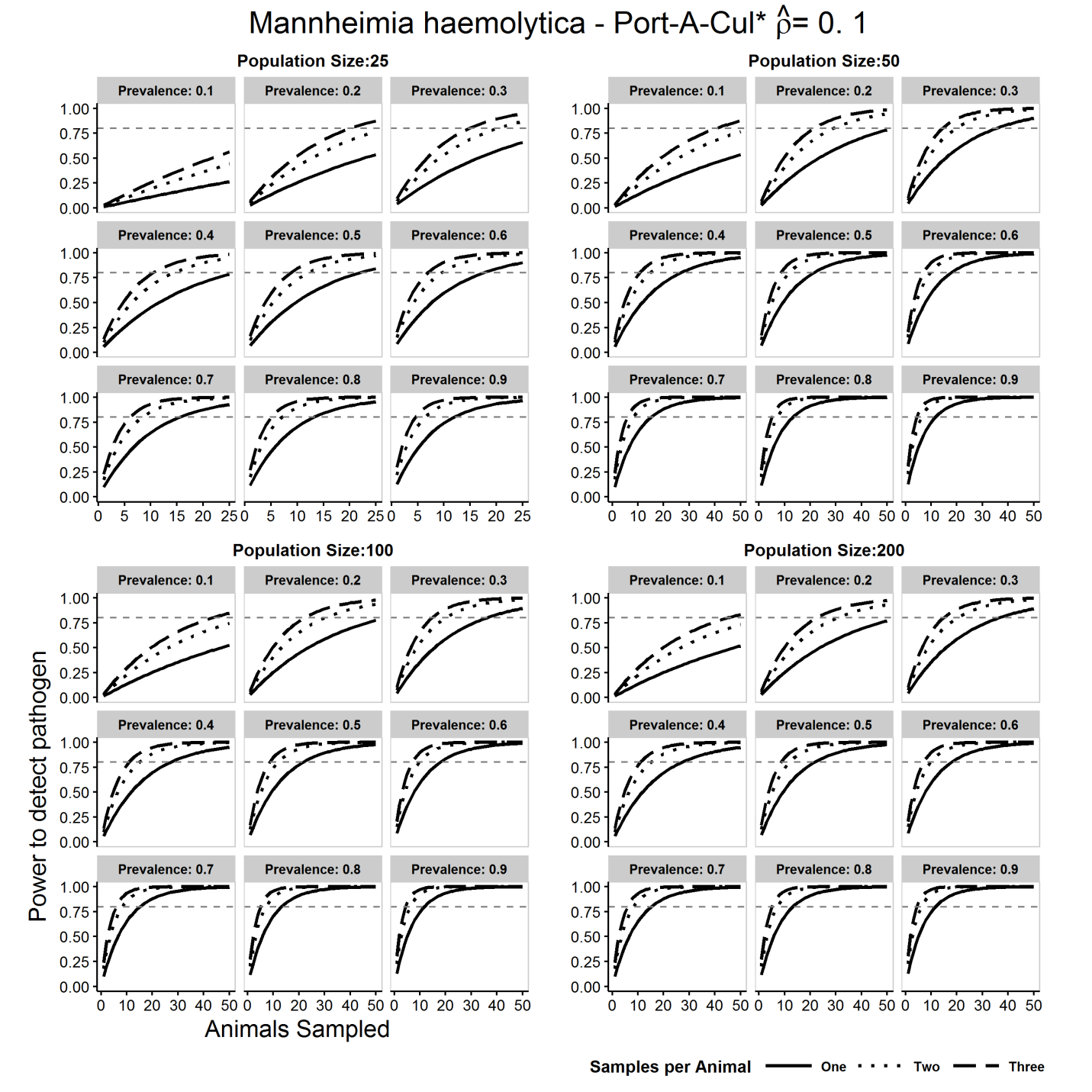


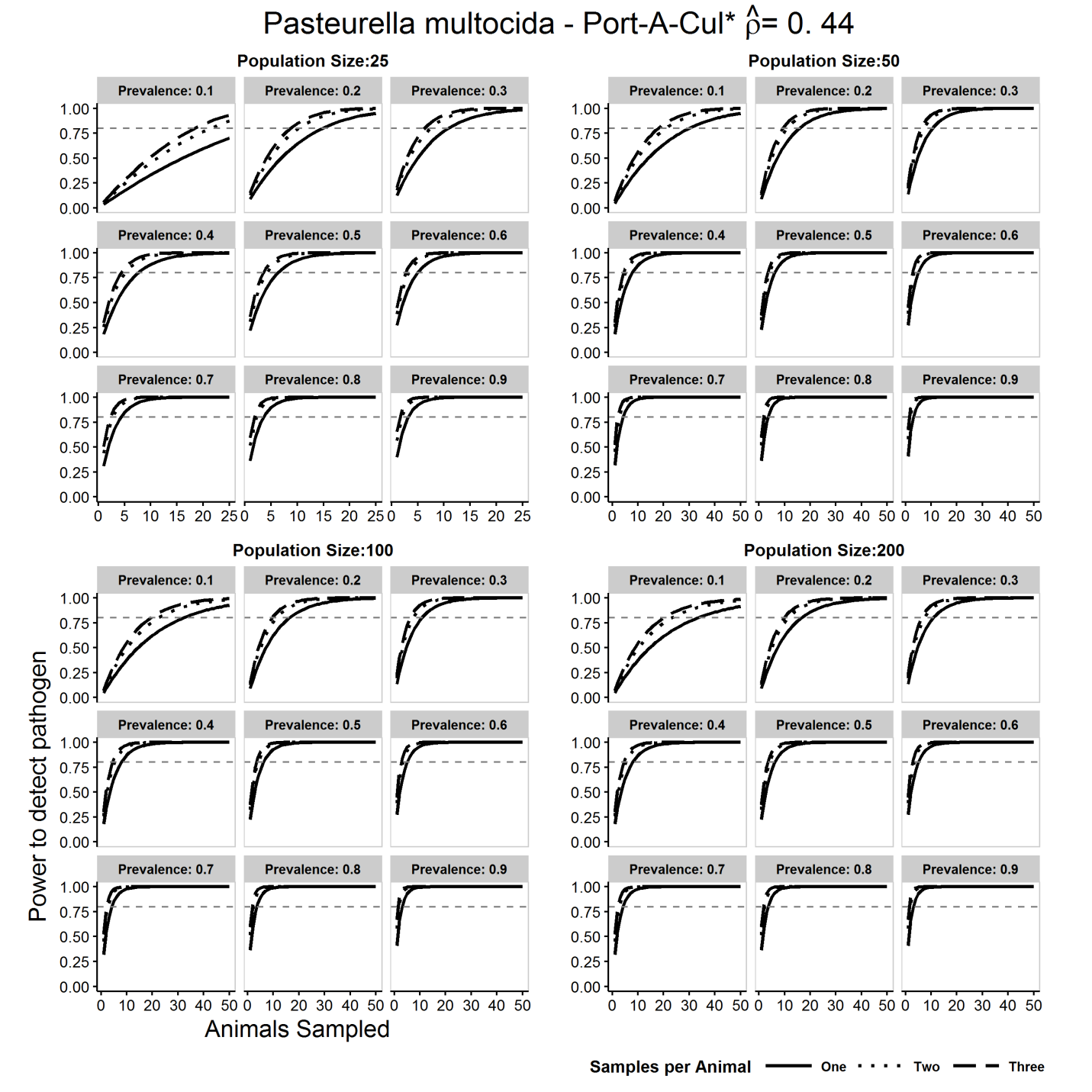


## *Plated Culture**


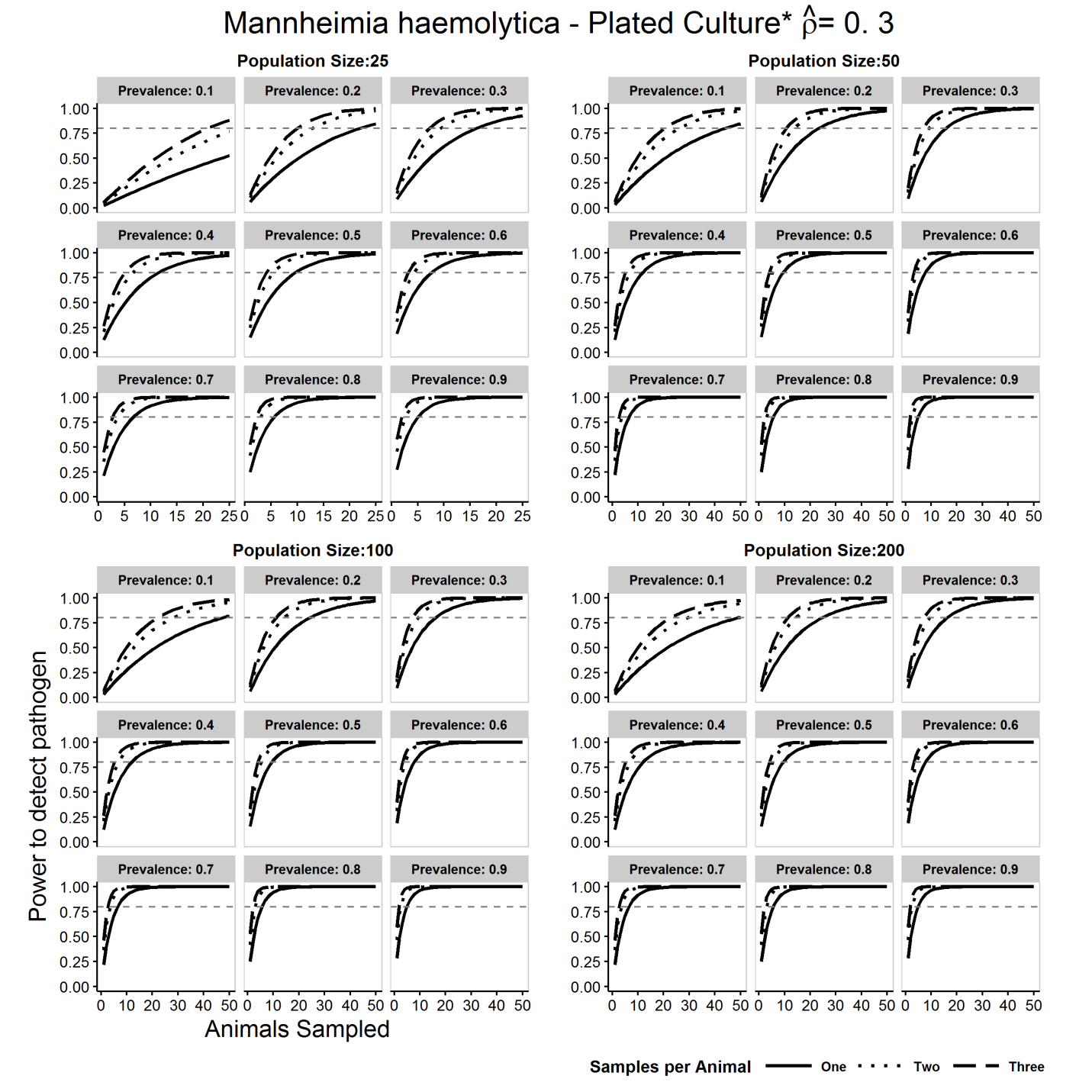


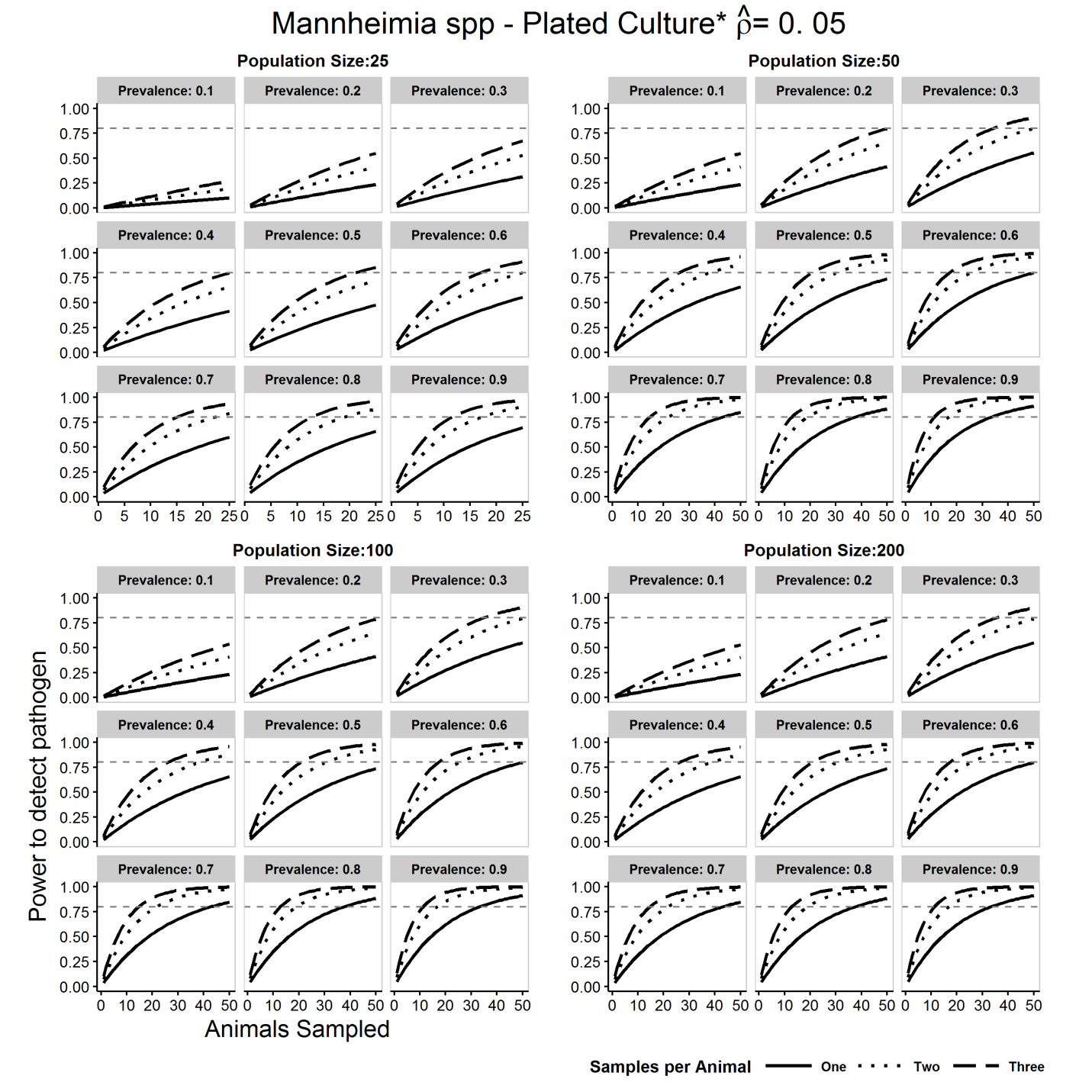


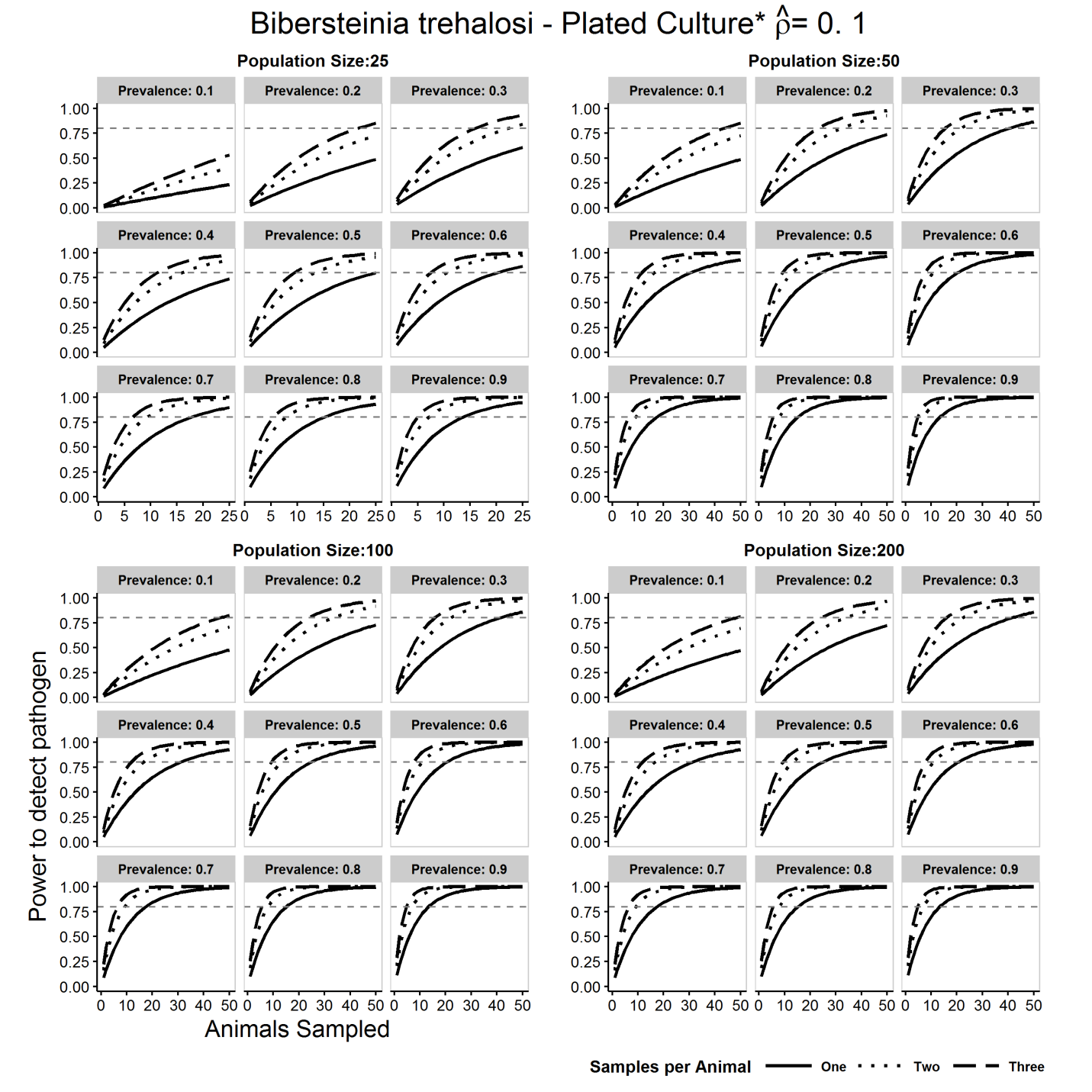


## *Plated PCR*


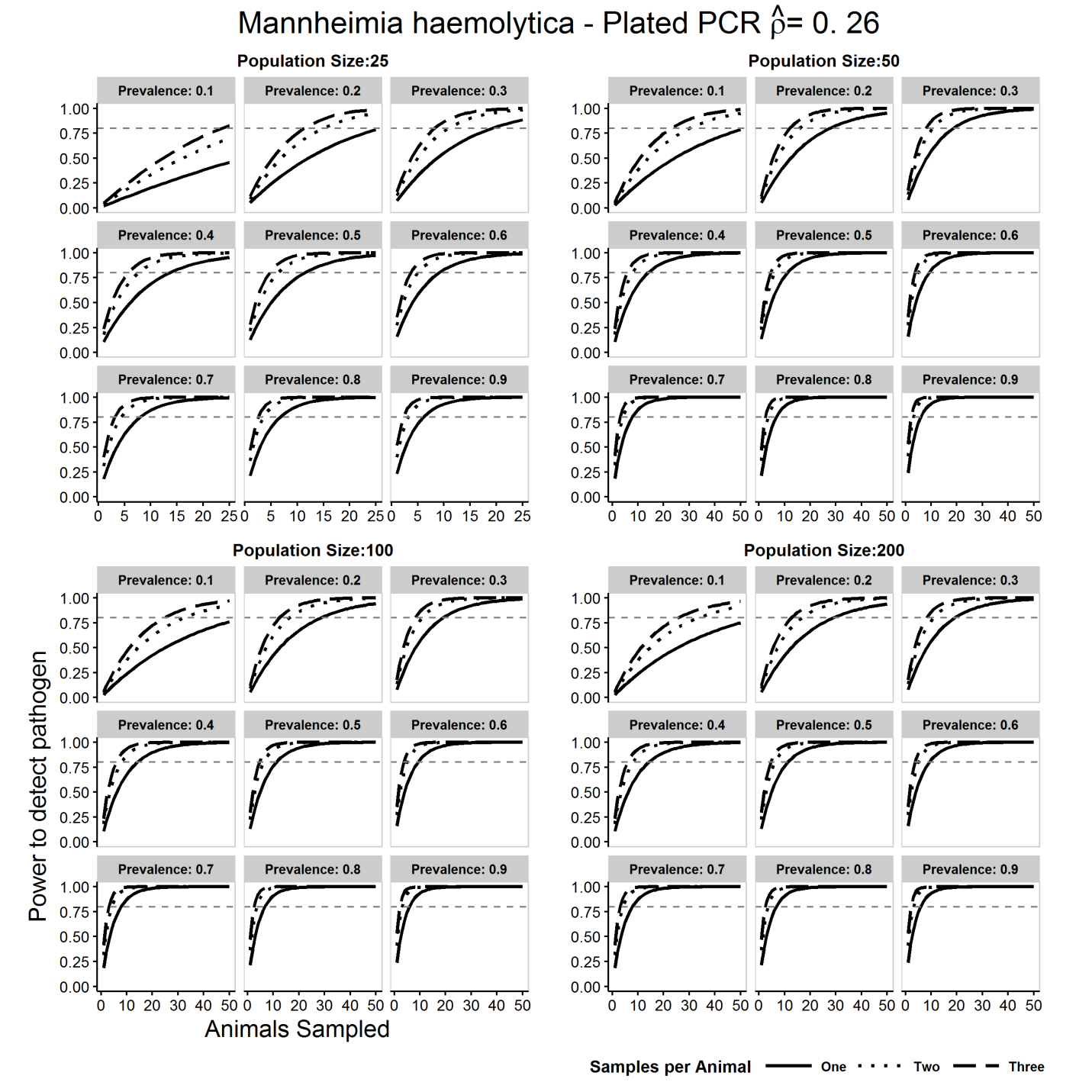


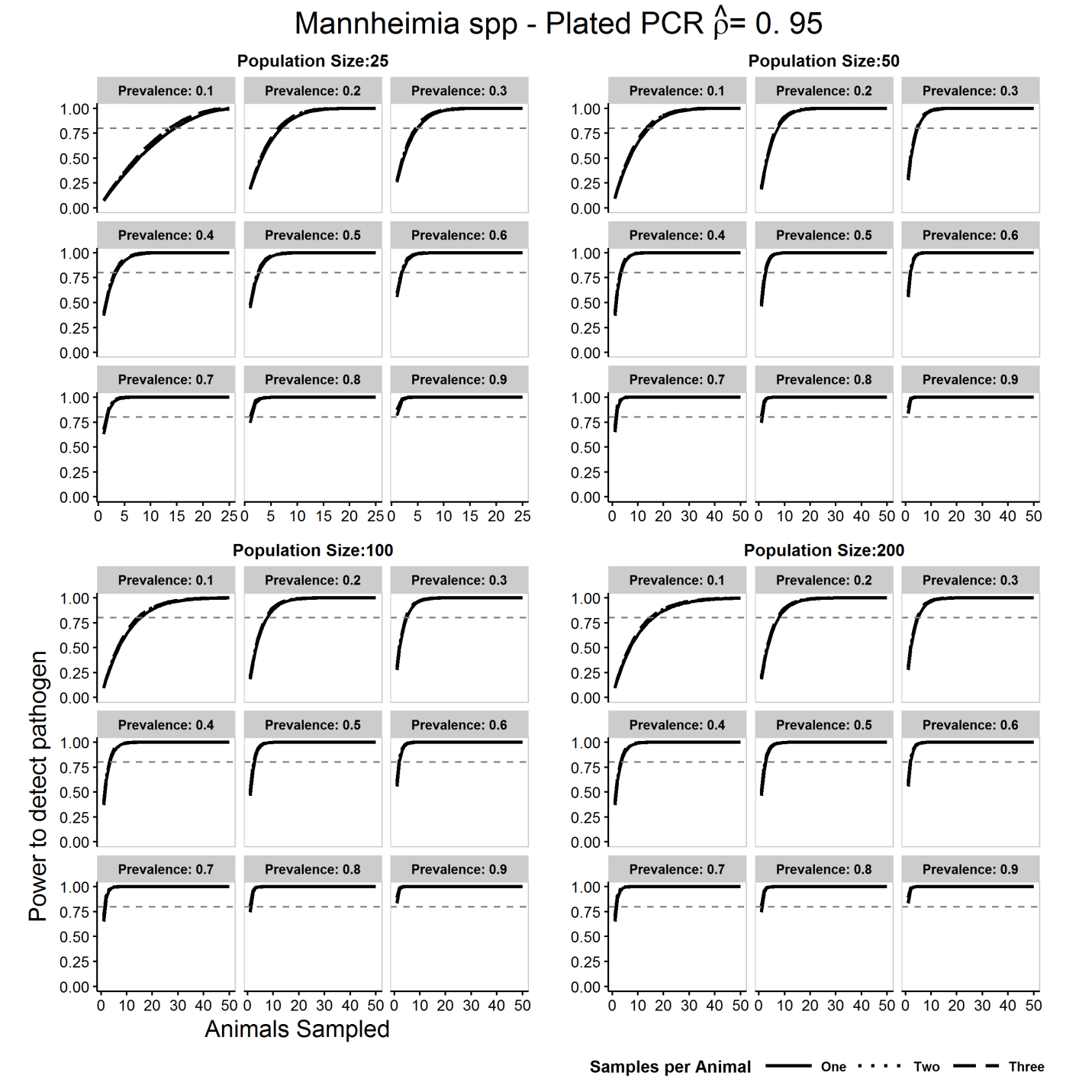


## *Wyoming*


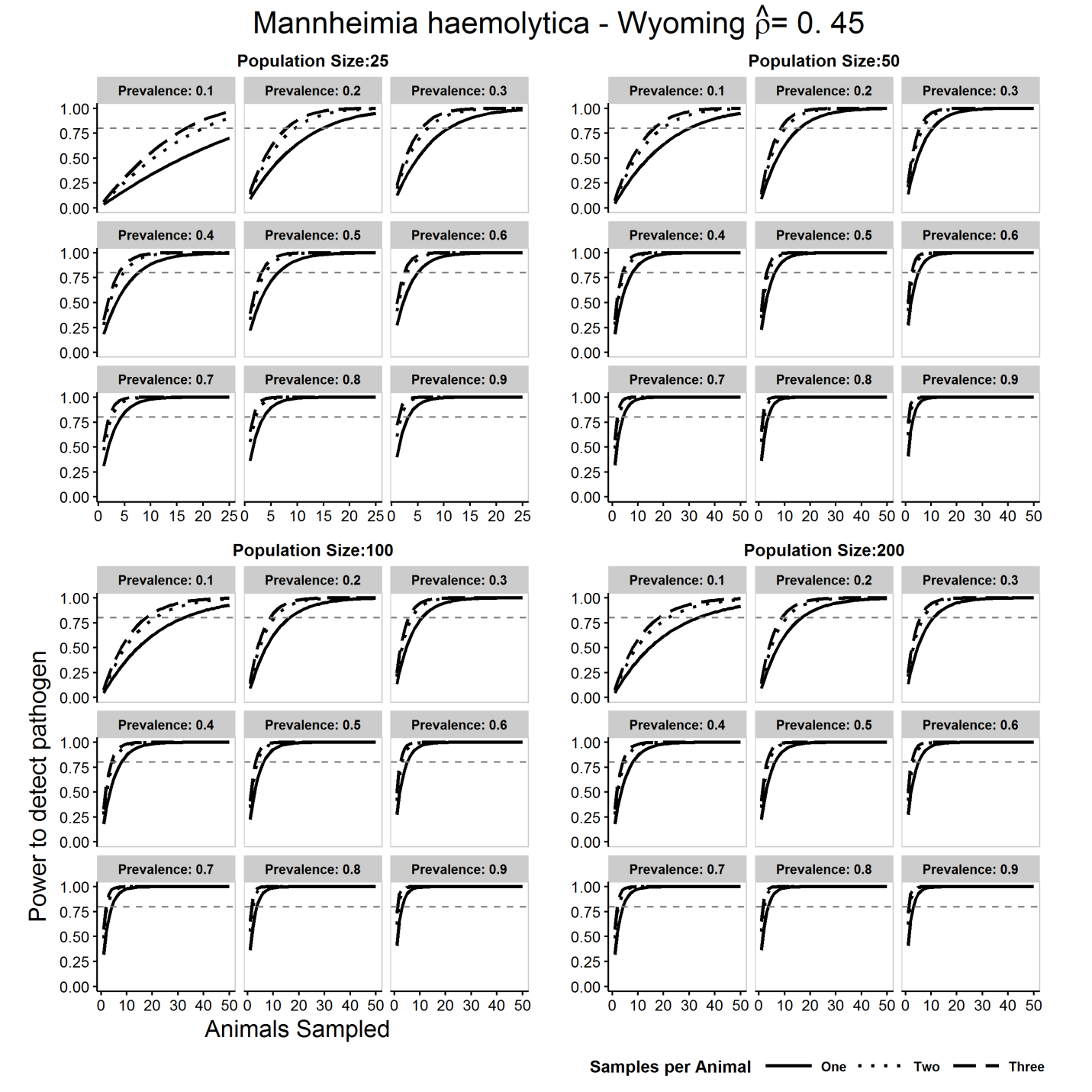


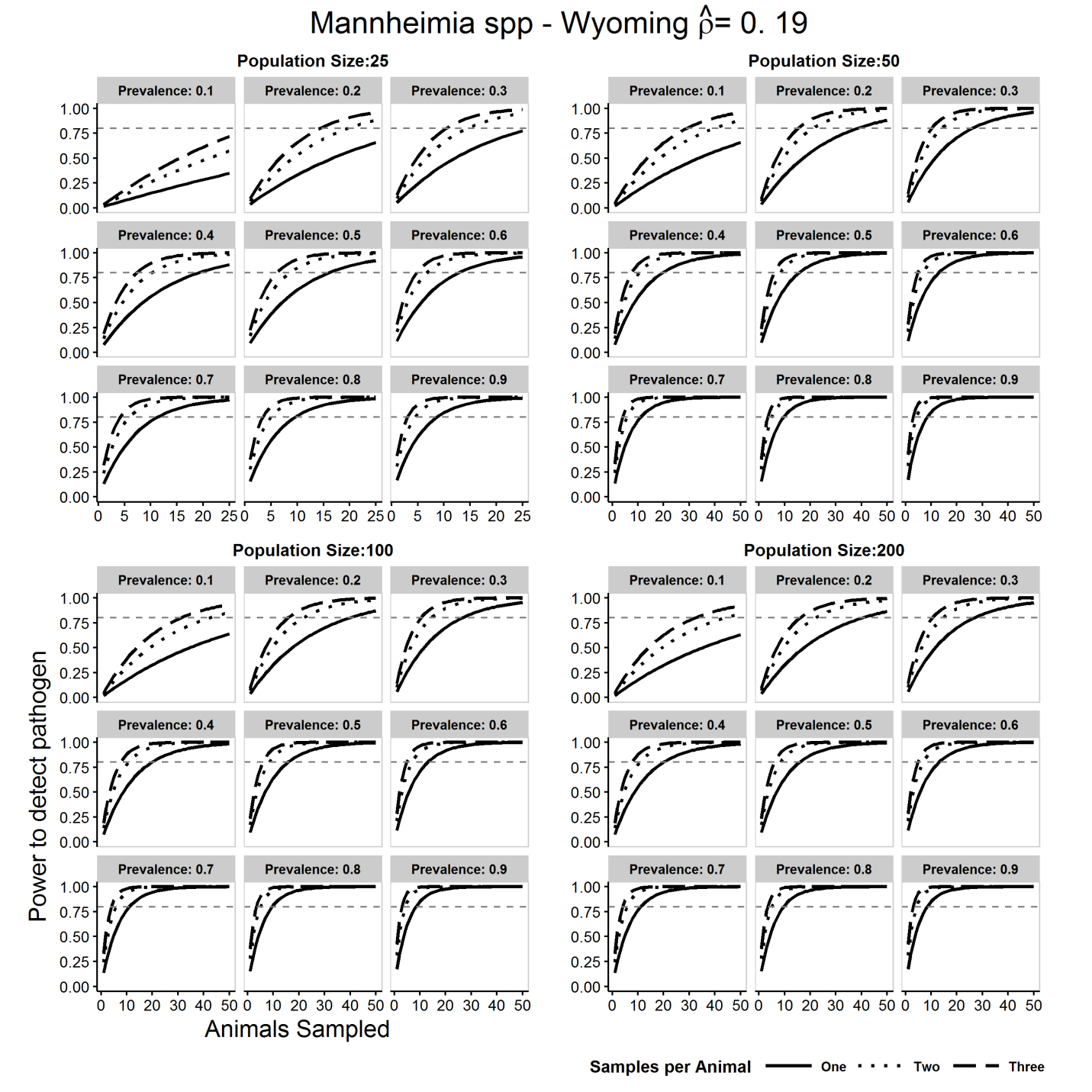


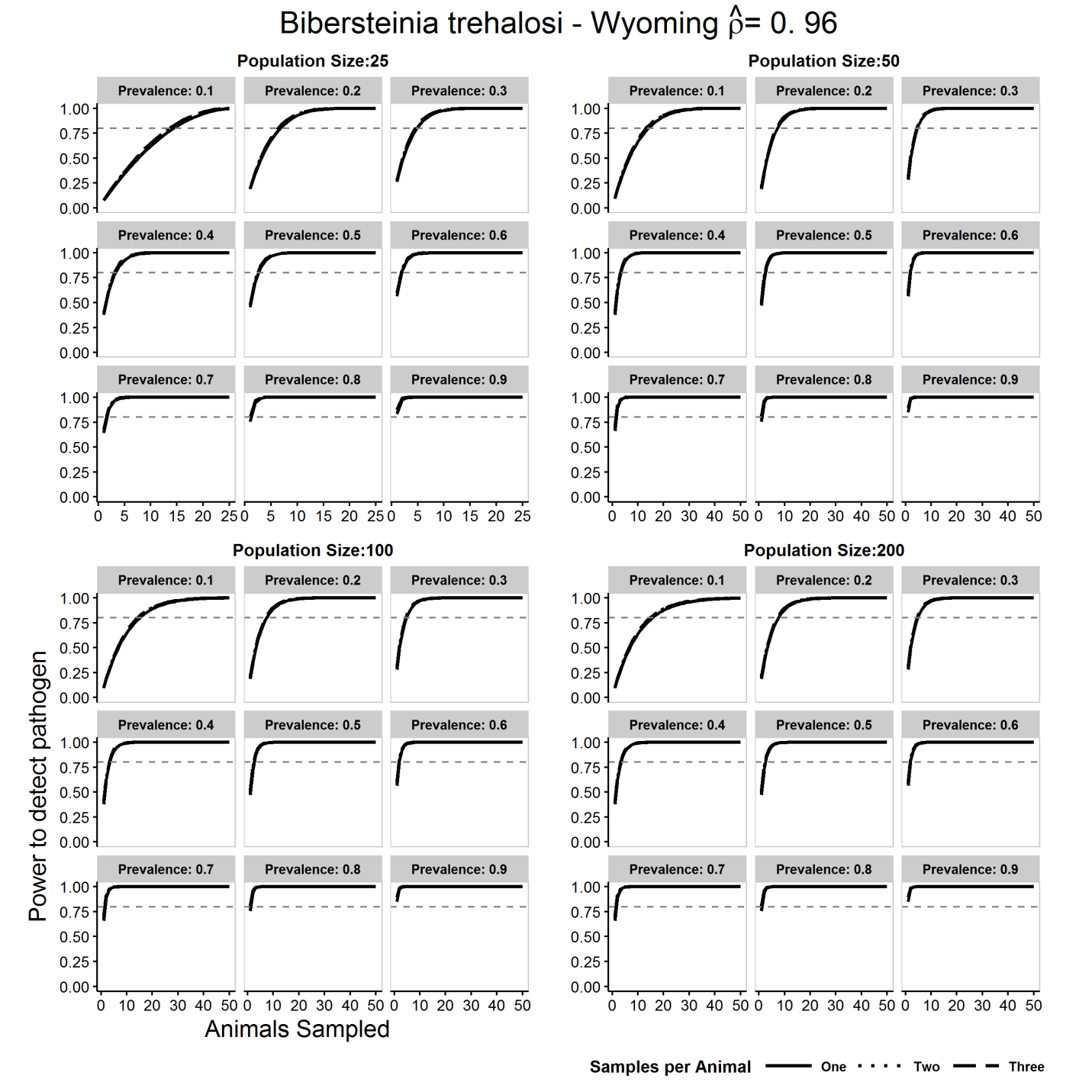


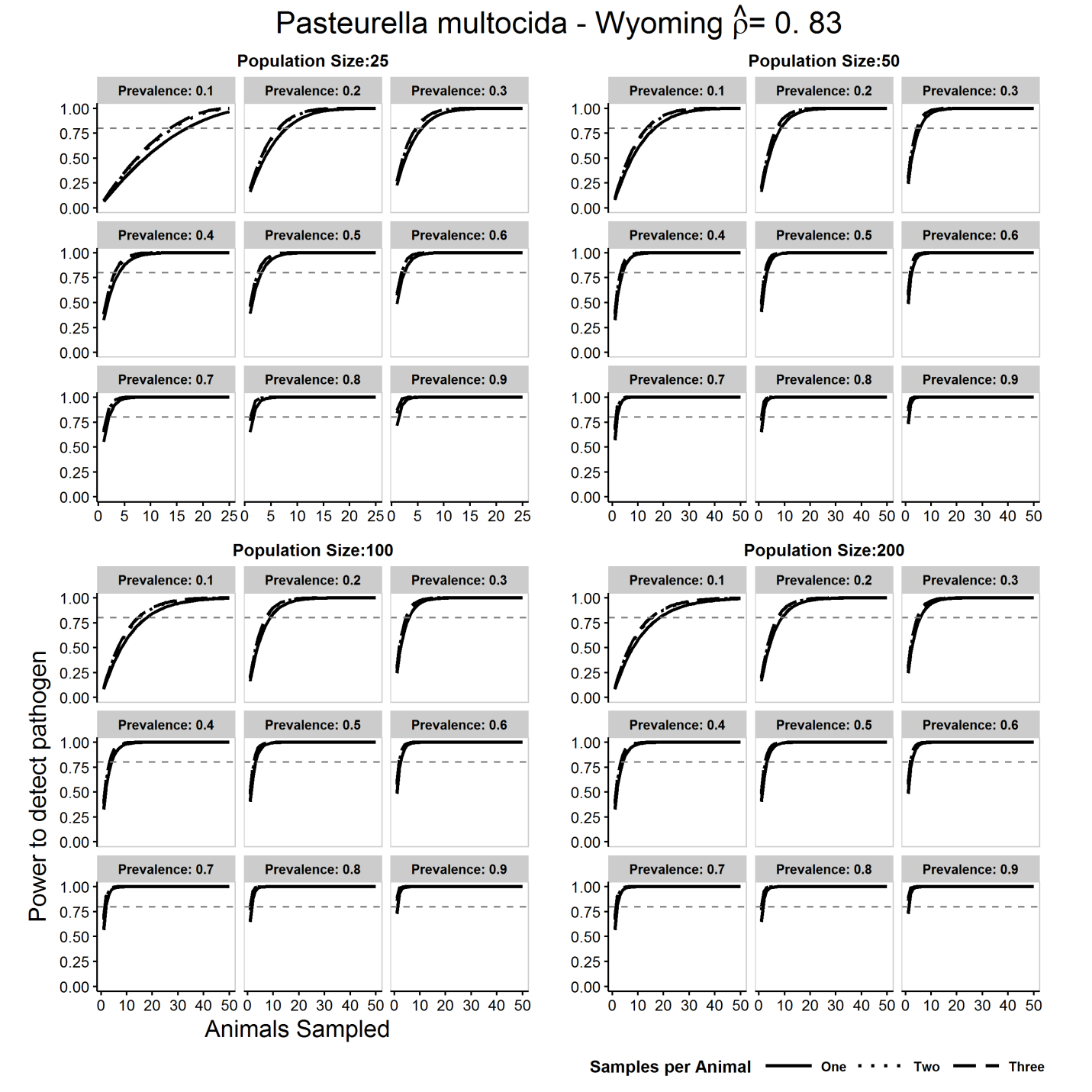

Supplement: S5 Appendix — (DOCX) [file pone.0180689.s005.docx]
